# Search for the Higgs boson produced in association with a *W* boson and decaying to four *b*-quarks via two spin-zero particles in *pp* collisions at 13 TeV with the ATLAS detector

**DOI:** 10.1140/epjc/s10052-016-4418-9

**Published:** 2016-11-05

**Authors:** M. Aaboud, G. Aad, B. Abbott, J. Abdallah, O. Abdinov, B. Abeloos, R. Aben, O. S. AbouZeid, N. L. Abraham, H. Abramowicz, H. Abreu, R. Abreu, Y. Abulaiti, B. S. Acharya, L. Adamczyk, D. L. Adams, J. Adelman, S. Adomeit, T. Adye, A. A. Affolder, T. Agatonovic-Jovin, J. Agricola, J. A. Aguilar-Saavedra, S. P. Ahlen, F. Ahmadov, G. Aielli, H. Akerstedt, T. P. A. Åkesson, A. V. Akimov, G. L. Alberghi, J. Albert, S. Albrand, M. J. Alconada Verzini, M. Aleksa, I. N. Aleksandrov, C. Alexa, G. Alexander, T. Alexopoulos, M. Alhroob, B. Ali, M. Aliev, G. Alimonti, J. Alison, S. P. Alkire, B. M. M. Allbrooke, B. W. Allen, P. P. Allport, A. Aloisio, A. Alonso, F. Alonso, C. Alpigiani, M. Alstaty, B. Alvarez Gonzalez, D. Álvarez Piqueras, M. G. Alviggi, B. T. Amadio, K. Amako, Y. Amaral Coutinho, C. Amelung, D. Amidei, S. P. Amor Dos Santos, A. Amorim, S. Amoroso, G. Amundsen, C. Anastopoulos, L. S. Ancu, N. Andari, T. Andeen, C. F. Anders, G. Anders, J. K. Anders, K. J. Anderson, A. Andreazza, V. Andrei, S. Angelidakis, I. Angelozzi, P. Anger, A. Angerami, F. Anghinolfi, A. V. Anisenkov, N. Anjos, A. Annovi, C. Antel, M. Antonelli, A. Antonov, F. Anulli, M. Aoki, L. Aperio Bella, G. Arabidze, Y. Arai, J. P. Araque, A. T. H. Arce, F. A. Arduh, J-F. Arguin, S. Argyropoulos, M. Arik, A. J. Armbruster, L. J. Armitage, O. Arnaez, H. Arnold, M. Arratia, O. Arslan, A. Artamonov, G. Artoni, S. Artz, S. Asai, N. Asbah, A. Ashkenazi, B. Åsman, L. Asquith, K. Assamagan, R. Astalos, M. Atkinson, N. B. Atlay, K. Augsten, G. Avolio, B. Axen, M. K. Ayoub, G. Azuelos, M. A. Baak, A. E. Baas, M. J. Baca, H. Bachacou, K. Bachas, M. Backes, M. Backhaus, P. Bagiacchi, P. Bagnaia, Y. Bai, J. T. Baines, O. K. Baker, E. M. Baldin, P. Balek, T. Balestri, F. Balli, W. K. Balunas, E. Banas, Sw. Banerjee, A. A. E. Bannoura, L. Barak, E. L. Barberio, D. Barberis, M. Barbero, T. Barillari, M-S Barisits, T. Barklow, N. Barlow, S. L. Barnes, B. M. Barnett, R. M. Barnett, Z. Barnovska-Blenessy, A. Baroncelli, G. Barone, A. J. Barr, L. Barranco Navarro, F. Barreiro, J. Barreiro Guimarães da Costa, R. Bartoldus, A. E. Barton, P. Bartos, A. Basalaev, A. Bassalat, R. L. Bates, S. J. Batista, J. R. Batley, M. Battaglia, M. Bauce, F. Bauer, H. S. Bawa, J. B. Beacham, M. D. Beattie, T. Beau, P. H. Beauchemin, P. Bechtle, H. P. Beck, K. Becker, M. Becker, M. Beckingham, C. Becot, A. J. Beddall, A. Beddall, V. A. Bednyakov, M. Bedognetti, C. P. Bee, L. J. Beemster, T. A. Beermann, M. Begel, J. K. Behr, C. Belanger-Champagne, A. S. Bell, G. Bella, L. Bellagamba, A. Bellerive, M. Bellomo, K. Belotskiy, O. Beltramello, N. L. Belyaev, O. Benary, D. Benchekroun, M. Bender, K. Bendtz, N. Benekos, Y. Benhammou, E. Benhar Noccioli, J. Benitez, D. P. Benjamin, J. R. Bensinger, S. Bentvelsen, L. Beresford, M. Beretta, D. Berge, E. Bergeaas Kuutmann, N. Berger, J. Beringer, S. Berlendis, N. R. Bernard, C. Bernius, F. U. Bernlochner, T. Berry, P. Berta, C. Bertella, G. Bertoli, F. Bertolucci, I. A. Bertram, C. Bertsche, D. Bertsche, G. J. Besjes, O. Bessidskaia Bylund, M. Bessner, N. Besson, C. Betancourt, A. Bethani, S. Bethke, A. J. Bevan, R. M. Bianchi, L. Bianchini, M. Bianco, O. Biebel, D. Biedermann, R. Bielski, N. V. Biesuz, M. Biglietti, J. Bilbao De Mendizabal, T. R. V. Billoud, H. Bilokon, M. Bindi, S. Binet, A. Bingul, C. Bini, S. Biondi, T. Bisanz, D. M. Bjergaard, C. W. Black, J. E. Black, K. M. Black, D. Blackburn, R. E. Blair, J. -B. Blanchard, T. Blazek, I. Bloch, C. Blocker, W. Blum, U. Blumenschein, S. Blunier, G. J. Bobbink, V. S. Bobrovnikov, S. S. Bocchetta, A. Bocci, C. Bock, M. Boehler, D. Boerner, J. A. Bogaerts, D. Bogavac, A. G. Bogdanchikov, C. Bohm, V. Boisvert, P. Bokan, T. Bold, A. S. Boldyrev, M. Bomben, M. Bona, M. Boonekamp, A. Borisov, G. Borissov, J. Bortfeldt, D. Bortoletto, V. Bortolotto, K. Bos, D. Boscherini, M. Bosman, J. D. Bossio Sola, J. Boudreau, J. Bouffard, E. V. Bouhova-Thacker, D. Boumediene, C. Bourdarios, S. K. Boutle, A. Boveia, J. Boyd, I. R. Boyko, J. Bracinik, A. Brandt, G. Brandt, O. Brandt, U. Bratzler, B. Brau, J. E. Brau, H. M. Braun, W. D. Breaden Madden, K. Brendlinger, A. J. Brennan, L. Brenner, R. Brenner, S. Bressler, T. M. Bristow, D. Britton, D. Britzger, F. M. Brochu, I. Brock, R. Brock, G. Brooijmans, T. Brooks, W. K. Brooks, J. Brosamer, E. Brost, J. H Broughton, P. A. Bruckman de Renstrom, D. Bruncko, R. Bruneliere, A. Bruni, G. Bruni, L. S. Bruni, BH Brunt, M. Bruschi, N. Bruscino, P. Bryant, L. Bryngemark, T. Buanes, Q. Buat, P. Buchholz, A. G. Buckley, I. A. Budagov, F. Buehrer, M. K. Bugge, O. Bulekov, D. Bullock, H. Burckhart, S. Burdin, C. D. Burgard, B. Burghgrave, K. Burka, S. Burke, I. Burmeister, J. T. P. Burr, E. Busato, D. Büscher, V. Büscher, P. Bussey, J. M. Butler, C. M. Buttar, J. M. Butterworth, P. Butti, W. Buttinger, A. Buzatu, A. R. Buzykaev, S. Cabrera Urbán, D. Caforio, V. M. Cairo, O. Cakir, N. Calace, P. Calafiura, A. Calandri, G. Calderini, P. Calfayan, G. Callea, L. P. Caloba, S. Calvente Lopez, D. Calvet, S. Calvet, T. P. Calvet, R. Camacho Toro, S. Camarda, P. Camarri, D. Cameron, R. Caminal Armadans, C. Camincher, S. Campana, M. Campanelli, A. Camplani, A. Campoverde, V. Canale, A. Canepa, M. Cano Bret, J. Cantero, T. Cao, M. D. M. Capeans Garrido, I. Caprini, M. Caprini, M. Capua, R. Caputo, R. M. Carbone, R. Cardarelli, F. Cardillo, I. Carli, T. Carli, G. Carlino, L. Carminati, S. Caron, E. Carquin, G. D. Carrillo-Montoya, J. R. Carter, J. Carvalho, D. Casadei, M. P. Casado, M. Casolino, D. W. Casper, E. Castaneda-Miranda, R. Castelijn, A. Castelli, V. Castillo Gimenez, N. F. Castro, A. Catinaccio, J. R. Catmore, A. Cattai, J. Caudron, V. Cavaliere, E. Cavallaro, D. Cavalli, M. Cavalli-Sforza, V. Cavasinni, F. Ceradini, L. Cerda Alberich, B. C. Cerio, A. S. Cerqueira, A. Cerri, L. Cerrito, F. Cerutti, M. Cerv, A. Cervelli, S. A. Cetin, A. Chafaq, D. Chakraborty, S. K. Chan, Y. L. Chan, P. Chang, J. D. Chapman, D. G. Charlton, A. Chatterjee, C. C. Chau, C. A. Chavez Barajas, S. Che, S. Cheatham, A. Chegwidden, S. Chekanov, S. V. Chekulaev, G. A. Chelkov, M. A. Chelstowska, C. Chen, H. Chen, K. Chen, S. Chen, S. Chen, X. Chen, Y. Chen, H. C. Cheng, H. J Cheng, Y. Cheng, A. Cheplakov, E. Cheremushkina, R. Cherkaoui El Moursli, V. Chernyatin, E. Cheu, L. Chevalier, V. Chiarella, G. Chiarelli, G. Chiodini, A. S. Chisholm, A. Chitan, M. V. Chizhov, K. Choi, A. R. Chomont, S. Chouridou, B. K. B. Chow, V. Christodoulou, D. Chromek-Burckhart, J. Chudoba, A. J. Chuinard, J. J. Chwastowski, L. Chytka, G. Ciapetti, A. K. Ciftci, D. Cinca, V. Cindro, I. A. Cioara, C. Ciocca, A. Ciocio, F. Cirotto, Z. H. Citron, M. Citterio, M. Ciubancan, A. Clark, B. L. Clark, M. R. Clark, P. J. Clark, R. N. Clarke, C. Clement, Y. Coadou, M. Cobal, A. Coccaro, J. Cochran, L. Colasurdo, B. Cole, A. P. Colijn, J. Collot, T. Colombo, G. Compostella, P. Conde Muiño, E. Coniavitis, S. H. Connell, I. A. Connelly, V. Consorti, S. Constantinescu, G. Conti, F. Conventi, M. Cooke, B. D. Cooper, A. M. Cooper-Sarkar, K. J. R. Cormier, T. Cornelissen, M. Corradi, F. Corriveau, A. Corso-Radu, A. Cortes-Gonzalez, G. Cortiana, G. Costa, M. J. Costa, D. Costanzo, G. Cottin, G. Cowan, B. E. Cox, K. Cranmer, S. J. Crawley, G. Cree, S. Crépé-Renaudin, F. Crescioli, W. A. Cribbs, M. Crispin Ortuzar, M. Cristinziani, V. Croft, G. Crosetti, A. Cueto, T. Cuhadar Donszelmann, J. Cummings, M. Curatolo, J. Cúth, H. Czirr, P. Czodrowski, G. D’amen, S. D’Auria, M. D’Onofrio, M. J. Da Cunha Sargedas De Sousa, C. Da Via, W. Dabrowski, T. Dado, T. Dai, O. Dale, F. Dallaire, C. Dallapiccola, M. Dam, J. R. Dandoy, N. P. Dang, A. C. Daniells, N. S. Dann, M. Danninger, M. Dano Hoffmann, V. Dao, G. Darbo, S. Darmora, J. Dassoulas, A. Dattagupta, W. Davey, C. David, T. Davidek, M. Davies, P. Davison, E. Dawe, I. Dawson, R. K. Daya-Ishmukhametova, K. De, R. de Asmundis, A. De Benedetti, S. De Castro, S. De Cecco, N. De Groot, P. de Jong, H. De la Torre, F. De Lorenzi, A. De Maria, D. De Pedis, A. De Salvo, U. De Sanctis, A. De Santo, J. B. De Vivie De Regie, W. J. Dearnaley, R. Debbe, C. Debenedetti, D. V. Dedovich, N. Dehghanian, I. Deigaard, M. Del Gaudio, J. Del Peso, T. Del Prete, D. Delgove, F. Deliot, C. M. Delitzsch, A. Dell’Acqua, L. Dell’Asta, M. Dell’Orso, M. Della Pietra, D. della Volpe, M. Delmastro, P. A. Delsart, D. A. DeMarco, S. Demers, M. Demichev, A. Demilly, S. P. Denisov, D. Denysiuk, D. Derendarz, J. E. Derkaoui, F. Derue, P. Dervan, K. Desch, C. Deterre, K. Dette, P. O. Deviveiros, A. Dewhurst, S. Dhaliwal, A. Di Ciaccio, L. Di Ciaccio, W. K. Di Clemente, C. Di Donato, A. Di Girolamo, B. Di Girolamo, B. Di Micco, R. Di Nardo, A. Di Simone, R. Di Sipio, D. Di Valentino, C. Diaconu, M. Diamond, F. A. Dias, M. A. Diaz, E. B. Diehl, J. Dietrich, S. Diglio, A. Dimitrievska, J. Dingfelder, P. Dita, S. Dita, F. Dittus, F. Djama, T. Djobava, J. I. Djuvsland, M. A. B. do Vale, D. Dobos, M. Dobre, C. Doglioni, J. Dolejsi, Z. Dolezal, M. Donadelli, S. Donati, P. Dondero, J. Donini, J. Dopke, A. Doria, M. T. Dova, A. T. Doyle, E. Drechsler, M. Dris, Y. Du, J. Duarte-Campderros, E. Duchovni, G. Duckeck, O. A. Ducu, D. Duda, A. Dudarev, A. Chr. Dudder, E. M. Duffield, L. Duflot, M. Dührssen, M. Dumancic, M. Dunford, H. Duran Yildiz, M. Düren, A. Durglishvili, D. Duschinger, B. Dutta, M. Dyndal, C. Eckardt, K. M. Ecker, R. C. Edgar, N. C. Edwards, T. Eifert, G. Eigen, K. Einsweiler, T. Ekelof, M. El Kacimi, V. Ellajosyula, M. Ellert, S. Elles, F. Ellinghaus, A. A. Elliot, N. Ellis, J. Elmsheuser, M. Elsing, D. Emeliyanov, Y. Enari, O. C. Endner, J. S. Ennis, J. Erdmann, A. Ereditato, G. Ernis, J. Ernst, M. Ernst, S. Errede, E. Ertel, M. Escalier, H. Esch, C. Escobar, B. Esposito, A. I. Etienvre, E. Etzion, H. Evans, A. Ezhilov, F. Fabbri, L. Fabbri, G. Facini, R. M. Fakhrutdinov, S. Falciano, R. J. Falla, J. Faltova, Y. Fang, M. Fanti, A. Farbin, A. Farilla, C. Farina, E. M. Farina, T. Farooque, S. Farrell, S. M. Farrington, P. Farthouat, F. Fassi, P. Fassnacht, D. Fassouliotis, M. Faucci Giannelli, A. Favareto, W. J. Fawcett, L. Fayard, O. L. Fedin, W. Fedorko, S. Feigl, L. Feligioni, C. Feng, E. J. Feng, H. Feng, A. B. Fenyuk, L. Feremenga, P. Fernandez Martinez, S. Fernandez Perez, J. Ferrando, A. Ferrari, P. Ferrari, R. Ferrari, D. E. Ferreira de Lima, A. Ferrer, D. Ferrere, C. Ferretti, A. Ferretto Parodi, F. Fiedler, A. Filipčič, M. Filipuzzi, F. Filthaut, M. Fincke-Keeler, K. D. Finelli, M. C. N. Fiolhais, L. Fiorini, A. Firan, A. Fischer, C. Fischer, J. Fischer, W. C. Fisher, N. Flaschel, I. Fleck, P. Fleischmann, G. T. Fletcher, R. R. M. Fletcher, T. Flick, A. Floderus, L. R. Flores Castillo, M. J. Flowerdew, G. T. Forcolin, A. Formica, A. Forti, A. G. Foster, D. Fournier, H. Fox, S. Fracchia, P. Francavilla, M. Franchini, D. Francis, L. Franconi, M. Franklin, M. Frate, M. Fraternali, D. Freeborn, S. M. Fressard-Batraneanu, F. Friedrich, D. Froidevaux, J. A. Frost, C. Fukunaga, E. Fullana Torregrosa, T. Fusayasu, J. Fuster, C. Gabaldon, O. Gabizon, A. Gabrielli, A. Gabrielli, G. P. Gach, S. Gadatsch, S. Gadomski, G. Gagliardi, L. G. Gagnon, P. Gagnon, C. Galea, B. Galhardo, E. J. Gallas, B. J. Gallop, P. Gallus, G. Galster, K. K. Gan, J. Gao, Y. Gao, Y. S. Gao, F. M. Garay Walls, C. García, J. E. García Navarro, M. Garcia-Sciveres, R. W. Gardner, N. Garelli, V. Garonne, A. Gascon Bravo, K. Gasnikova, C. Gatti, A. Gaudiello, G. Gaudio, L. Gauthier, I. L. Gavrilenko, C. Gay, G. Gaycken, E. N. Gazis, Z. Gecse, C. N. P. Gee, Ch. Geich-Gimbel, M. Geisen, M. P. Geisler, C. Gemme, M. H. Genest, C. Geng, S. Gentile, C. Gentsos, S. George, D. Gerbaudo, A. Gershon, S. Ghasemi, H. Ghazlane, M. Ghneimat, B. Giacobbe, S. Giagu, P. Giannetti, B. Gibbard, S. M. Gibson, M. Gignac, M. Gilchriese, T. P. S. Gillam, D. Gillberg, G. Gilles, D. M. Gingrich, N. Giokaris, M. P. Giordani, F. M. Giorgi, F. M. Giorgi, P. F. Giraud, P. Giromini, D. Giugni, F. Giuli, C. Giuliani, M. Giulini, B. K. Gjelsten, S. Gkaitatzis, I. Gkialas, E. L. Gkougkousis, L. K. Gladilin, C. Glasman, J. Glatzer, P. C. F. Glaysher, A. Glazov, M. Goblirsch-Kolb, J. Godlewski, S. Goldfarb, T. Golling, D. Golubkov, A. Gomes, R. Gonçalo, J. Goncalves Pinto Firmino Da Costa, G. Gonella, L. Gonella, A. Gongadze, S. González de la Hoz, G. Gonzalez Parra, S. Gonzalez-Sevilla, L. Goossens, P. A. Gorbounov, H. A. Gordon, I. Gorelov, B. Gorini, E. Gorini, A. Gorišek, E. Gornicki, A. T. Goshaw, C. Gössling, M. I. Gostkin, C. R. Goudet, D. Goujdami, A. G. Goussiou, N. Govender, E. Gozani, L. Graber, I. Grabowska-Bold, P. O. J. Gradin, P. Grafström, J. Gramling, E. Gramstad, S. Grancagnolo, V. Gratchev, P. M. Gravila, H. M. Gray, E. Graziani, Z. D. Greenwood, C. Grefe, K. Gregersen, I. M. Gregor, P. Grenier, K. Grevtsov, J. Griffiths, A. A. Grillo, K. Grimm, S. Grinstein, Ph. Gris, J. -F. Grivaz, S. Groh, J. P. Grohs, E. Gross, J. Grosse-Knetter, G. C. Grossi, Z. J. Grout, L. Guan, W. Guan, J. Guenther, F. Guescini, D. Guest, O. Gueta, E. Guido, T. Guillemin, S. Guindon, U. Gul, C. Gumpert, J. Guo, Y. Guo, R. Gupta, S. Gupta, G. Gustavino, P. Gutierrez, N. G. Gutierrez Ortiz, C. Gutschow, C. Guyot, C. Gwenlan, C. B. Gwilliam, A. Haas, C. Haber, H. K. Hadavand, N. Haddad, A. Hadef, S. Hageböck, Z. Hajduk, H. Hakobyan, M. Haleem, J. Haley, G. Halladjian, G. D. Hallewell, K. Hamacher, P. Hamal, K. Hamano, A. Hamilton, G. N. Hamity, P. G. Hamnett, L. Han, K. Hanagaki, K. Hanawa, M. Hance, B. Haney, P. Hanke, R. Hanna, J. B. Hansen, J. D. Hansen, M. C. Hansen, P. H. Hansen, K. Hara, A. S. Hard, T. Harenberg, F. Hariri, S. Harkusha, R. D. Harrington, P. F. Harrison, F. Hartjes, N. M. Hartmann, M. Hasegawa, Y. Hasegawa, A. Hasib, S. Hassani, S. Haug, R. Hauser, L. Hauswald, M. Havranek, C. M. Hawkes, R. J. Hawkings, D. Hayakawa, D. Hayden, C. P. Hays, J. M. Hays, H. S. Hayward, S. J. Haywood, S. J. Head, T. Heck, V. Hedberg, L. Heelan, S. Heim, T. Heim, B. Heinemann, J. J. Heinrich, L. Heinrich, C. Heinz, J. Hejbal, L. Helary, S. Hellman, C. Helsens, J. Henderson, R. C. W. Henderson, Y. Heng, S. Henkelmann, A. M. Henriques Correia, S. Henrot-Versille, G. H. Herbert, V. Herget, Y. Hernández Jiménez, G. Herten, R. Hertenberger, L. Hervas, G. G. Hesketh, N. P. Hessey, J. W. Hetherly, R. Hickling, E. Higón-Rodriguez, E. Hill, J. C. Hill, K. H. Hiller, S. J. Hillier, I. Hinchliffe, E. Hines, R. R. Hinman, M. Hirose, D. Hirschbuehl, J. Hobbs, N. Hod, M. C. Hodgkinson, P. Hodgson, A. Hoecker, M. R. Hoeferkamp, F. Hoenig, D. Hohn, T. R. Holmes, M. Homann, T. M. Hong, B. H. Hooberman, W. H. Hopkins, Y. Horii, A. J. Horton, J-Y. Hostachy, S. Hou, A. Hoummada, J. Howarth, M. Hrabovsky, I. Hristova, J. Hrivnac, T. Hryn’ova, A. Hrynevich, C. Hsu, P. J. Hsu, S. -C. Hsu, D. Hu, Q. Hu, S. Hu, Y. Huang, Z. Hubacek, F. Hubaut, F. Huegging, T. B. Huffman, E. W. Hughes, G. Hughes, M. Huhtinen, P. Huo, N. Huseynov, J. Huston, J. Huth, G. Iacobucci, G. Iakovidis, I. Ibragimov, L. Iconomidou-Fayard, E. Ideal, Z. Idrissi, P. Iengo, O. Igonkina, T. Iizawa, Y. Ikegami, M. Ikeno, Y. Ilchenko, D. Iliadis, N. Ilic, T. Ince, G. Introzzi, P. Ioannou, M. Iodice, K. Iordanidou, V. Ippolito, N. Ishijima, M. Ishino, M. Ishitsuka, R. Ishmukhametov, C. Issever, S. Istin, F. Ito, J. M. Iturbe Ponce, R. Iuppa, W. Iwanski, H. Iwasaki, J. M. Izen, V. Izzo, S. Jabbar, B. Jackson, P. Jackson, V. Jain, K. B. Jakobi, K. Jakobs, S. Jakobsen, T. Jakoubek, D. O. Jamin, D. K. Jana, E. Jansen, R. Jansky, J. Janssen, M. Janus, G. Jarlskog, N. Javadov, T. Javůrek, F. Jeanneau, L. Jeanty, G. -Y. Jeng, D. Jennens, P. Jenni, C. Jeske, S. Jézéquel, H. Ji, J. Jia, H. Jiang, Y. Jiang, S. Jiggins, J. Jimenez Pena, S. Jin, A. Jinaru, O. Jinnouchi, H. Jivan, P. Johansson, K. A. Johns, W. J. Johnson, K. Jon-And, G. Jones, R. W. L. Jones, S. Jones, T. J. Jones, J. Jongmanns, P. M. Jorge, J. Jovicevic, X. Ju, A. Juste Rozas, M. K. Köhler, A. Kaczmarska, M. Kado, H. Kagan, M. Kagan, S. J. Kahn, T. Kaji, E. Kajomovitz, C. W. Kalderon, A. Kaluza, S. Kama, A. Kamenshchikov, N. Kanaya, S. Kaneti, L. Kanjir, V. A. Kantserov, J. Kanzaki, B. Kaplan, L. S. Kaplan, A. Kapliy, D. Kar, K. Karakostas, A. Karamaoun, N. Karastathis, M. J. Kareem, E. Karentzos, M. Karnevskiy, S. N. Karpov, Z. M. Karpova, K. Karthik, V. Kartvelishvili, A. N. Karyukhin, K. Kasahara, L. Kashif, R. D. Kass, A. Kastanas, Y. Kataoka, C. Kato, A. Katre, J. Katzy, K. Kawade, K. Kawagoe, T. Kawamoto, G. Kawamura, V. F. Kazanin, R. Keeler, R. Kehoe, J. S. Keller, J. J. Kempster, H. Keoshkerian, O. Kepka, B. P. Kerševan, S. Kersten, R. A. Keyes, M. Khader, F. Khalil-zada, A. Khanov, A. G. Kharlamov, T. J. Khoo, V. Khovanskiy, E. Khramov, J. Khubua, S. Kido, C. R. Kilby, H. Y. Kim, S. H. Kim, Y. K. Kim, N. Kimura, O. M. Kind, B. T. King, M. King, J. Kirk, A. E. Kiryunin, T. Kishimoto, D. Kisielewska, F. Kiss, K. Kiuchi, O. Kivernyk, E. Kladiva, M. H. Klein, M. Klein, U. Klein, K. Kleinknecht, P. Klimek, A. Klimentov, R. Klingenberg, J. A. Klinger, T. Klioutchnikova, E. -E. Kluge, P. Kluit, S. Kluth, J. Knapik, E. Kneringer, E. B. F. G. Knoops, A. Knue, A. Kobayashi, D. Kobayashi, T. Kobayashi, M. Kobel, M. Kocian, P. Kodys, N. M. Koehler, T. Koffas, E. Koffeman, T. Koi, H. Kolanoski, M. Kolb, I. Koletsou, A. A. Komar, Y. Komori, T. Kondo, N. Kondrashova, K. Köneke, A. C. König, T. Kono, R. Konoplich, N. Konstantinidis, R. Kopeliansky, S. Koperny, L. Köpke, A. K. Kopp, K. Korcyl, K. Kordas, A. Korn, A. A. Korol, I. Korolkov, E. V. Korolkova, O. Kortner, S. Kortner, T. Kosek, V. V. Kostyukhin, A. Kotwal, A. Kourkoumeli-Charalampidi, C. Kourkoumelis, V. Kouskoura, A. B. Kowalewska, R. Kowalewski, T. Z. Kowalski, C. Kozakai, W. Kozanecki, A. S. Kozhin, V. A. Kramarenko, G. Kramberger, D. Krasnopevtsev, M. W. Krasny, A. Krasznahorkay, A. Kravchenko, M. Kretz, J. Kretzschmar, K. Kreutzfeldt, P. Krieger, K. Krizka, K. Kroeninger, H. Kroha, J. Kroll, J. Kroseberg, J. Krstic, U. Kruchonak, H. Krüger, N. Krumnack, A. Kruse, M. C. Kruse, M. Kruskal, T. Kubota, H. Kucuk, S. Kuday, J. T. Kuechler, S. Kuehn, A. Kugel, F. Kuger, A. Kuhl, T. Kuhl, V. Kukhtin, R. Kukla, Y. Kulchitsky, S. Kuleshov, M. Kuna, T. Kunigo, A. Kupco, H. Kurashige, Y. A. Kurochkin, V. Kus, E. S. Kuwertz, M. Kuze, J. Kvita, T. Kwan, D. Kyriazopoulos, A. La Rosa, J. L. La Rosa Navarro, L. La Rotonda, C. Lacasta, F. Lacava, J. Lacey, H. Lacker, D. Lacour, V. R. Lacuesta, E. Ladygin, R. Lafaye, B. Laforge, T. Lagouri, S. Lai, S. Lammers, W. Lampl, E. Lançon, U. Landgraf, M. P. J. Landon, M. C. Lanfermann, V. S. Lang, J. C. Lange, A. J. Lankford, F. Lanni, K. Lantzsch, A. Lanza, S. Laplace, C. Lapoire, J. F. Laporte, T. Lari, F. Lasagni Manghi, M. Lassnig, P. Laurelli, W. Lavrijsen, A. T. Law, P. Laycock, T. Lazovich, M. Lazzaroni, B. Le, O. Le Dortz, E. Le Guirriec, E. P. Le Quilleuc, M. LeBlanc, T. LeCompte, F. Ledroit-Guillon, C. A. Lee, S. C. Lee, L. Lee, B. Lefebvre, G. Lefebvre, M. Lefebvre, F. Legger, C. Leggett, A. Lehan, G. Lehmann Miotto, X. Lei, W. A. Leight, A. G. Leister, M. A. L. Leite, R. Leitner, D. Lellouch, B. Lemmer, K. J. C. Leney, T. Lenz, B. Lenzi, R. Leone, S. Leone, C. Leonidopoulos, S. Leontsinis, G. Lerner, C. Leroy, A. A. J. Lesage, C. G. Lester, M. Levchenko, J. Levêque, D. Levin, L. J. Levinson, M. Levy, D. Lewis, A. M. Leyko, M. Leyton, B. Li, C. Li, H. Li, H. L. Li, L. Li, L. Li, Q. Li, S. Li, X. Li, Y. Li, Z. Liang, B. Liberti, A. Liblong, P. Lichard, K. Lie, J. Liebal, W. Liebig, A. Limosani, S. C. Lin, T. H. Lin, B. E. Lindquist, A. E. Lionti, E. Lipeles, A. Lipniacka, M. Lisovyi, T. M. Liss, A. Lister, A. M. Litke, B. Liu, D. Liu, H. Liu, H. Liu, J. Liu, J. B. Liu, K. Liu, L. Liu, M. Liu, M. Liu, Y. L. Liu, Y. Liu, M. Livan, A. Lleres, J. Llorente Merino, S. L. Lloyd, F. Lo Sterzo, E. M. Lobodzinska, P. Loch, W. S. Lockman, F. K. Loebinger, A. E. Loevschall-Jensen, K. M. Loew, A. Loginov, T. Lohse, K. Lohwasser, M. Lokajicek, B. A. Long, J. D. Long, R. E. Long, L. Longo, K. A. Looper, L. Lopes, D. Lopez Mateos, B. Lopez Paredes, I. Lopez Paz, A. Lopez Solis, J. Lorenz, N. Lorenzo Martinez, M. Losada, P. J. Lösel, X. Lou, A. Lounis, J. Love, P. A. Love, H. Lu, N. Lu, H. J. Lubatti, C. Luci, A. Lucotte, C. Luedtke, F. Luehring, W. Lukas, L. Luminari, O. Lundberg, B. Lund-Jensen, P. M. Luzi, D. Lynn, R. Lysak, E. Lytken, V. Lyubushkin, H. Ma, L. L. Ma, Y. Ma, G. Maccarrone, A. Macchiolo, C. M. Macdonald, B. Maček, J. Machado Miguens, D. Madaffari, R. Madar, H. J. Maddocks, W. F. Mader, A. Madsen, J. Maeda, S. Maeland, T. Maeno, A. Maevskiy, E. Magradze, J. Mahlstedt, C. Maiani, C. Maidantchik, A. A. Maier, T. Maier, A. Maio, S. Majewski, Y. Makida, N. Makovec, B. Malaescu, Pa. Malecki, V. P. Maleev, F. Malek, U. Mallik, D. Malon, C. Malone, S. Maltezos, S. Malyukov, J. Mamuzic, G. Mancini, B. Mandelli, L. Mandelli, I. Mandić, J. Maneira, L. Manhaes de Andrade Filho, J. Manjarres Ramos, A. Mann, A. Manousos, B. Mansoulie, J. D. Mansour, R. Mantifel, M. Mantoani, S. Manzoni, L. Mapelli, G. Marceca, L. March, G. Marchiori, M. Marcisovsky, M. Marjanovic, D. E. Marley, F. Marroquim, S. P. Marsden, Z. Marshall, S. Marti-Garcia, B. Martin, T. A. Martin, V. J. Martin, B. Martin dit Latour, M. Martinez, V. I. Martinez Outschoorn, S. Martin-Haugh, V. S. Martoiu, A. C. Martyniuk, M. Marx, A. Marzin, L. Masetti, T. Mashimo, R. Mashinistov, J. Masik, A. L. Maslennikov, I. Massa, L. Massa, P. Mastrandrea, A. Mastroberardino, T. Masubuchi, P. Mättig, J. Mattmann, J. Maurer, S. J. Maxfield, D. A. Maximov, R. Mazini, S. M. Mazza, N. C. Mc Fadden, G. Mc Goldrick, S. P. Mc Kee, A. McCarn, R. L. McCarthy, T. G. McCarthy, L. I. McClymont, E. F. McDonald, J. A. Mcfayden, G. Mchedlidze, S. J. McMahon, R. A. McPherson, M. Medinnis, S. Meehan, S. Mehlhase, A. Mehta, K. Meier, C. Meineck, B. Meirose, D. Melini, B. R. Mellado Garcia, M. Melo, F. Meloni, A. Mengarelli, S. Menke, E. Meoni, S. Mergelmeyer, P. Mermod, L. Merola, C. Meroni, F. S. Merritt, A. Messina, J. Metcalfe, A. S. Mete, C. Meyer, C. Meyer, J-P. Meyer, J. Meyer, H. Meyer Zu Theenhausen, F. Miano, R. P. Middleton, S. Miglioranzi, L. Mijović, G. Mikenberg, M. Mikestikova, M. Mikuž, M. Milesi, A. Milic, D. W. Miller, C. Mills, A. Milov, D. A. Milstead, A. A. Minaenko, Y. Minami, I. A. Minashvili, A. I. Mincer, B. Mindur, M. Mineev, Y. Ming, L. M. Mir, K. P. Mistry, T. Mitani, J. Mitrevski, V. A. Mitsou, A. Miucci, P. S. Miyagawa, J. U. Mjörnmark, T. Moa, K. Mochizuki, S. Mohapatra, S. Molander, R. Moles-Valls, R. Monden, M. C. Mondragon, K. Mönig, J. Monk, E. Monnier, A. Montalbano, J. Montejo Berlingen, F. Monticelli, S. Monzani, R. W. Moore, N. Morange, D. Moreno, M. Moreno Llácer, P. Morettini, S. Morgenstern, D. Mori, T. Mori, M. Morii, M. Morinaga, V. Morisbak, S. Moritz, A. K. Morley, G. Mornacchi, J. D. Morris, S. S. Mortensen, L. Morvaj, M. Mosidze, J. Moss, K. Motohashi, R. Mount, E. Mountricha, S. V. Mouraviev, E. J. W. Moyse, S. Muanza, R. D. Mudd, F. Mueller, J. Mueller, R. S. P. Mueller, T. Mueller, D. Muenstermann, P. Mullen, G. A. Mullier, F. J. Munoz Sanchez, J. A. Murillo Quijada, W. J. Murray, H. Musheghyan, M. Muškinja, A. G. Myagkov, M. Myska, B. P. Nachman, O. Nackenhorst, K. Nagai, R. Nagai, K. Nagano, Y. Nagasaka, K. Nagata, M. Nagel, E. Nagy, A. M. Nairz, Y. Nakahama, K. Nakamura, T. Nakamura, I. Nakano, H. Namasivayam, R. F. Naranjo Garcia, R. Narayan, D. I. Narrias Villar, I. Naryshkin, T. Naumann, G. Navarro, R. Nayyar, H. A. Neal, P. Yu. Nechaeva, T. J. Neep, A. Negri, M. Negrini, S. Nektarijevic, C. Nellist, A. Nelson, S. Nemecek, P. Nemethy, A. A. Nepomuceno, M. Nessi, M. S. Neubauer, M. Neumann, R. M. Neves, P. Nevski, P. R. Newman, D. H. Nguyen, T. Nguyen Manh, R. B. Nickerson, R. Nicolaidou, J. Nielsen, A. Nikiforov, V. Nikolaenko, I. Nikolic-Audit, K. Nikolopoulos, J. K. Nilsen, P. Nilsson, Y. Ninomiya, A. Nisati, R. Nisius, T. Nobe, M. Nomachi, I. Nomidis, T. Nooney, S. Norberg, M. Nordberg, N. Norjoharuddeen, O. Novgorodova, S. Nowak, M. Nozaki, L. Nozka, K. Ntekas, E. Nurse, F. Nuti, F. O’grady, D. C. O’Neil, A. A. O’Rourke, V. O’Shea, F. G. Oakham, H. Oberlack, T. Obermann, J. Ocariz, A. Ochi, I. Ochoa, J. P. Ochoa-Ricoux, S. Oda, S. Odaka, H. Ogren, A. Oh, S. H. Oh, C. C. Ohm, H. Ohman, H. Oide, H. Okawa, Y. Okumura, T. Okuyama, A. Olariu, L. F. Oleiro Seabra, S. A. Olivares Pino, D. Oliveira Damazio, A. Olszewski, J. Olszowska, A. Onofre, K. Onogi, P. U. E. Onyisi, M. J. Oreglia, Y. Oren, D. Orestano, N. Orlando, R. S. Orr, B. Osculati, R. Ospanov, G. Otero y Garzon, H. Otono, M. Ouchrif, F. Ould-Saada, A. Ouraou, K. P. Oussoren, Q. Ouyang, M. Owen, R. E. Owen, V. E. Ozcan, N. Ozturk, K. Pachal, A. Pacheco Pages, L. Pacheco Rodriguez, C. Padilla Aranda, M. Pagáčová, S. Pagan Griso, F. Paige, P. Pais, K. Pajchel, G. Palacino, S. Palazzo, S. Palestini, M. Palka, D. Pallin, E. St. Panagiotopoulou, C. E. Pandini, J. G. Panduro Vazquez, P. Pani, S. Panitkin, D. Pantea, L. Paolozzi, Th. D. Papadopoulou, K. Papageorgiou, A. Paramonov, D. Paredes Hernandez, A. J. Parker, M. A. Parker, K. A. Parker, F. Parodi, J. A. Parsons, U. Parzefall, V. R. Pascuzzi, E. Pasqualucci, S. Passaggio, Fr. Pastore, G. Pásztor, S. Pataraia, J. R. Pater, T. Pauly, J. Pearce, B. Pearson, L. E. Pedersen, M. Pedersen, S. Pedraza Lopez, R. Pedro, S. V. Peleganchuk, O. Penc, C. Peng, H. Peng, J. Penwell, B. S. Peralva, M. M. Perego, D. V. Perepelitsa, E. Perez Codina, L. Perini, H. Pernegger, S. Perrella, R. Peschke, V. D. Peshekhonov, K. Peters, R. F. Y. Peters, B. A. Petersen, T. C. Petersen, E. Petit, A. Petridis, C. Petridou, P. Petroff, E. Petrolo, M. Petrov, F. Petrucci, N. E. Pettersson, A. Peyaud, R. Pezoa, P. W. Phillips, G. Piacquadio, E. Pianori, A. Picazio, E. Piccaro, M. Piccinini, M. A. Pickering, R. Piegaia, J. E. Pilcher, A. D. Pilkington, A. W. J. Pin, M. Pinamonti, J. L. Pinfold, A. Pingel, S. Pires, H. Pirumov, M. Pitt, L. Plazak, M. -A. Pleier, V. Pleskot, E. Plotnikova, P. Plucinski, D. Pluth, R. Poettgen, L. Poggioli, D. Pohl, G. Polesello, A. Poley, A. Policicchio, R. Polifka, A. Polini, C. S. Pollard, V. Polychronakos, K. Pommès, L. Pontecorvo, B. G. Pope, G. A. Popeneciu, A. Poppleton, S. Pospisil, K. Potamianos, I. N. Potrap, C. J. Potter, C. T. Potter, G. Poulard, J. Poveda, V. Pozdnyakov, M. E. Pozo Astigarraga, P. Pralavorio, A. Pranko, S. Prell, D. Price, L. E. Price, M. Primavera, S. Prince, K. Prokofiev, F. Prokoshin, S. Protopopescu, J. Proudfoot, M. Przybycien, D. Puddu, M. Purohit, P. Puzo, J. Qian, G. Qin, Y. Qin, A. Quadt, W. B. Quayle, M. Queitsch-Maitland, D. Quilty, S. Raddum, V. Radeka, V. Radescu, S. K. Radhakrishnan, P. Radloff, P. Rados, F. Ragusa, G. Rahal, J. A. Raine, S. Rajagopalan, M. Rammensee, C. Rangel-Smith, M. G. Ratti, F. Rauscher, S. Rave, T. Ravenscroft, I. Ravinovich, M. Raymond, A. L. Read, N. P. Readioff, M. Reale, D. M. Rebuzzi, A. Redelbach, G. Redlinger, R. Reece, K. Reeves, L. Rehnisch, J. Reichert, H. Reisin, C. Rembser, H. Ren, M. Rescigno, S. Resconi, O. L. Rezanova, P. Reznicek, R. Rezvani, R. Richter, S. Richter, E. Richter-Was, O. Ricken, M. Ridel, P. Rieck, C. J. Riegel, J. Rieger, O. Rifki, M. Rijssenbeek, A. Rimoldi, M. Rimoldi, L. Rinaldi, B. Ristić, E. Ritsch, I. Riu, F. Rizatdinova, E. Rizvi, C. Rizzi, S. H. Robertson, A. Robichaud-Veronneau, D. Robinson, J. E. M. Robinson, A. Robson, C. Roda, Y. Rodina, A. Rodriguez Perez, D. Rodriguez Rodriguez, S. Roe, C. S. Rogan, O. Røhne, A. Romaniouk, M. Romano, S. M. Romano Saez, E. Romero Adam, N. Rompotis, M. Ronzani, L. Roos, E. Ros, S. Rosati, K. Rosbach, P. Rose, O. Rosenthal, N. -A. Rosien, V. Rossetti, E. Rossi, L. P. Rossi, J. H. N. Rosten, R. Rosten, M. Rotaru, I. Roth, J. Rothberg, D. Rousseau, C. R. Royon, A. Rozanov, Y. Rozen, X. Ruan, F. Rubbo, M. S. Rudolph, F. Rühr, A. Ruiz-Martinez, Z. Rurikova, N. A. Rusakovich, A. Ruschke, H. L. Russell, J. P. Rutherfoord, N. Ruthmann, Y. F. Ryabov, M. Rybar, G. Rybkin, S. Ryu, A. Ryzhov, G. F. Rzehorz, A. F. Saavedra, G. Sabato, S. Sacerdoti, H. F-W. Sadrozinski, R. Sadykov, F. Safai Tehrani, P. Saha, M. Sahinsoy, M. Saimpert, T. Saito, H. Sakamoto, Y. Sakurai, G. Salamanna, A. Salamon, J. E. Salazar Loyola, D. Salek, P. H. Sales De Bruin, D. Salihagic, A. Salnikov, J. Salt, D. Salvatore, F. Salvatore, A. Salvucci, A. Salzburger, D. Sammel, D. Sampsonidis, J. Sánchez, V. Sanchez Martinez, A. Sanchez Pineda, H. Sandaker, R. L. Sandbach, H. G. Sander, M. Sandhoff, C. Sandoval, R. Sandstroem, D. P. C. Sankey, M. Sannino, A. Sansoni, C. Santoni, R. Santonico, H. Santos, I. Santoyo Castillo, K. Sapp, A. Sapronov, J. G. Saraiva, B. Sarrazin, O. Sasaki, Y. Sasaki, K. Sato, G. Sauvage, E. Sauvan, G. Savage, P. Savard, N. Savic, C. Sawyer, L. Sawyer, J. Saxon, C. Sbarra, A. Sbrizzi, T. Scanlon, D. A. Scannicchio, M. Scarcella, V. Scarfone, J. Schaarschmidt, P. Schacht, B. M. Schachtner, D. Schaefer, L. Schaefer, R. Schaefer, J. Schaeffer, S. Schaepe, S. Schaetzel, U. Schäfer, A. C. Schaffer, D. Schaile, R. D. Schamberger, V. Scharf, V. A. Schegelsky, D. Scheirich, M. Schernau, C. Schiavi, S. Schier, C. Schillo, M. Schioppa, S. Schlenker, K. R. Schmidt-Sommerfeld, K. Schmieden, C. Schmitt, S. Schmitt, S. Schmitz, B. Schneider, U. Schnoor, L. Schoeffel, A. Schoening, B. D. Schoenrock, E. Schopf, M. Schott, J. Schovancova, S. Schramm, M. Schreyer, N. Schuh, A. Schulte, M. J. Schultens, H. -C. Schultz-Coulon, H. Schulz, M. Schumacher, B. A. Schumm, Ph. Schune, A. Schwartzman, T. A. Schwarz, H. Schweiger, Ph. Schwemling, R. Schwienhorst, J. Schwindling, T. Schwindt, G. Sciolla, F. Scuri, F. Scutti, J. Searcy, P. Seema, S. C. Seidel, A. Seiden, F. Seifert, J. M. Seixas, G. Sekhniaidze, K. Sekhon, S. J. Sekula, D. M. Seliverstov, N. Semprini-Cesari, C. Serfon, L. Serin, L. Serkin, M. Sessa, R. Seuster, H. Severini, T. Sfiligoj, F. Sforza, A. Sfyrla, E. Shabalina, N. W. Shaikh, L. Y. Shan, R. Shang, J. T. Shank, M. Shapiro, P. B. Shatalov, K. Shaw, S. M. Shaw, A. Shcherbakova, C. Y. Shehu, P. Sherwood, L. Shi, S. Shimizu, C. O. Shimmin, M. Shimojima, M. Shiyakova, A. Shmeleva, D. Shoaleh Saadi, M. J. Shochet, S. Shojaii, S. Shrestha, E. Shulga, M. A. Shupe, P. Sicho, A. M. Sickles, P. E. Sidebo, O. Sidiropoulou, D. Sidorov, A. Sidoti, F. Siegert, Dj. Sijacki, J. Silva, S. B. Silverstein, V. Simak, Lj. Simic, S. Simion, E. Simioni, B. Simmons, D. Simon, M. Simon, P. Sinervo, N. B. Sinev, M. Sioli, G. Siragusa, S. Yu. Sivoklokov, J. Sjölin, M. B. Skinner, H. P. Skottowe, P. Skubic, M. Slater, T. Slavicek, M. Slawinska, K. Sliwa, R. Slovak, V. Smakhtin, B. H. Smart, L. Smestad, J. Smiesko, S. Yu. Smirnov, Y. Smirnov, L. N. Smirnova, O. Smirnova, M. N. K. Smith, R. W. Smith, M. Smizanska, K. Smolek, A. A. Snesarev, S. Snyder, R. Sobie, F. Socher, A. Soffer, D. A. Soh, G. Sokhrannyi, C. A. Solans Sanchez, M. Solar, E. Yu. Soldatov, U. Soldevila, A. A. Solodkov, A. Soloshenko, O. V. Solovyanov, V. Solovyev, P. Sommer, H. Son, H. Y. Song, A. Sood, A. Sopczak, V. Sopko, V. Sorin, D. Sosa, C. L. Sotiropoulou, R. Soualah, A. M. Soukharev, D. South, B. C. Sowden, S. Spagnolo, M. Spalla, M. Spangenberg, F. Spanò, D. Sperlich, F. Spettel, R. Spighi, G. Spigo, L. A. Spiller, M. Spousta, R. D. St. Denis, A. Stabile, R. Stamen, S. Stamm, E. Stanecka, R. W. Stanek, C. Stanescu, M. Stanescu-Bellu, M. M. Stanitzki, S. Stapnes, E. A. Starchenko, G. H. Stark, J. Stark, P. Staroba, P. Starovoitov, S. Stärz, R. Staszewski, P. Steinberg, B. Stelzer, H. J. Stelzer, O. Stelzer-Chilton, H. Stenzel, G. A. Stewart, J. A. Stillings, M. C. Stockton, M. Stoebe, G. Stoicea, P. Stolte, S. Stonjek, A. R. Stradling, A. Straessner, M. E. Stramaglia, J. Strandberg, S. Strandberg, A. Strandlie, M. Strauss, P. Strizenec, R. Ströhmer, D. M. Strom, R. Stroynowski, A. Strubig, S. A. Stucci, B. Stugu, N. A. Styles, D. Su, J. Su, S. Suchek, Y. Sugaya, M. Suk, V. V. Sulin, S. Sultansoy, T. Sumida, S. Sun, X. Sun, J. E. Sundermann, K. Suruliz, G. Susinno, M. R. Sutton, S. Suzuki, M. Svatos, M. Swiatlowski, I. Sykora, T. Sykora, D. Ta, C. Taccini, K. Tackmann, J. Taenzer, A. Taffard, R. Tafirout, N. Taiblum, H. Takai, R. Takashima, T. Takeshita, Y. Takubo, M. Talby, A. A. Talyshev, K. G. Tan, J. Tanaka, M. Tanaka, R. Tanaka, S. Tanaka, B. B. Tannenwald, S. Tapia Araya, S. Tapprogge, S. Tarem, G. F. Tartarelli, P. Tas, M. Tasevsky, T. Tashiro, E. Tassi, A. Tavares Delgado, Y. Tayalati, A. C. Taylor, G. N. Taylor, P. T. E. Taylor, W. Taylor, F. A. Teischinger, P. Teixeira-Dias, K. K. Temming, D. Temple, H. Ten Kate, P. K. Teng, J. J. Teoh, F. Tepel, S. Terada, K. Terashi, J. Terron, S. Terzo, M. Testa, R. J. Teuscher, T. Theveneaux-Pelzer, J. P. Thomas, J. Thomas-Wilsker, E. N. Thompson, P. D. Thompson, A. S. Thompson, L. A. Thomsen, E. Thomson, M. Thomson, M. J. Tibbetts, R. E. Ticse Torres, V. O. Tikhomirov, Yu. A. Tikhonov, S. Timoshenko, P. Tipton, S. Tisserant, K. Todome, T. Todorov, S. Todorova-Nova, J. Tojo, S. Tokár, K. Tokushuku, E. Tolley, L. Tomlinson, M. Tomoto, L. Tompkins, K. Toms, B. Tong, E. Torrence, H. Torres, E. Torró Pastor, J. Toth, F. Touchard, D. R. Tovey, T. Trefzger, A. Tricoli, I. M. Trigger, S. Trincaz-Duvoid, M. F. Tripiana, W. Trischuk, B. Trocmé, A. Trofymov, C. Troncon, M. Trottier-McDonald, M. Trovatelli, L. Truong, M. Trzebinski, A. Trzupek, J. C-L. Tseng, P. V. Tsiareshka, G. Tsipolitis, N. Tsirintanis, S. Tsiskaridze, V. Tsiskaridze, E. G. Tskhadadze, K. M. Tsui, I. I. Tsukerman, V. Tsulaia, S. Tsuno, D. Tsybychev, Y. Tu, A. Tudorache, V. Tudorache, A. N. Tuna, S. A. Tupputi, S. Turchikhin, D. Turecek, D. Turgeman, R. Turra, A. J. Turvey, P. M. Tuts, M. Tyndel, G. Ucchielli, I. Ueda, M. Ughetto, F. Ukegawa, G. Unal, A. Undrus, G. Unel, F. C. Ungaro, Y. Unno, C. Unverdorben, J. Urban, P. Urquijo, P. Urrejola, G. Usai, A. Usanova, L. Vacavant, V. Vacek, B. Vachon, C. Valderanis, E. Valdes Santurio, N. Valencic, S. Valentinetti, A. Valero, L. Valery, S. Valkar, J. A. Valls Ferrer, W. Van Den Wollenberg, P. C. Van Der Deijl, H. van der Graaf, N. van Eldik, P. van Gemmeren, J. Van Nieuwkoop, I. van Vulpen, M. C. van Woerden, M. Vanadia, W. Vandelli, R. Vanguri, A. Vaniachine, P. Vankov, G. Vardanyan, R. Vari, E. W. Varnes, T. Varol, D. Varouchas, A. Vartapetian, K. E. Varvell, J. G. Vasquez, F. Vazeille, T. Vazquez Schroeder, J. Veatch, V. Veeraraghavan, L. M. Veloce, F. Veloso, S. Veneziano, A. Ventura, M. Venturi, N. Venturi, A. Venturini, V. Vercesi, M. Verducci, W. Verkerke, J. C. Vermeulen, A. Vest, M. C. Vetterli, O. Viazlo, I. Vichou, T. Vickey, O. E. Vickey Boeriu, G. H. A. Viehhauser, S. Viel, L. Vigani, M. Villa, M. Villaplana Perez, E. Vilucchi, M. G. Vincter, V. B. Vinogradov, C. Vittori, I. Vivarelli, S. Vlachos, M. Vlasak, M. Vogel, P. Vokac, G. Volpi, M. Volpi, H. von der Schmitt, E. von Toerne, V. Vorobel, K. Vorobev, M. Vos, R. Voss, J. H. Vossebeld, N. Vranjes, M. Vranjes Milosavljevic, V. Vrba, M. Vreeswijk, R. Vuillermet, I. Vukotic, Z. Vykydal, P. Wagner, W. Wagner, H. Wahlberg, S. Wahrmund, J. Wakabayashi, J. Walder, R. Walker, W. Walkowiak, V. Wallangen, C. Wang, C. Wang, F. Wang, H. Wang, H. Wang, J. Wang, J. Wang, K. Wang, R. Wang, S. M. Wang, T. Wang, T. Wang, W. Wang, X. Wang, C. Wanotayaroj, A. Warburton, C. P. Ward, D. R. Wardrope, A. Washbrook, P. M. Watkins, A. T. Watson, M. F. Watson, G. Watts, S. Watts, B. M. Waugh, S. Webb, M. S. Weber, S. W. Weber, J. S. Webster, A. R. Weidberg, B. Weinert, J. Weingarten, C. Weiser, H. Weits, P. S. Wells, T. Wenaus, T. Wengler, S. Wenig, N. Wermes, M. Werner, M. D. Werner, P. Werner, M. Wessels, J. Wetter, K. Whalen, N. L. Whallon, A. M. Wharton, A. White, M. J. White, R. White, D. Whiteson, F. J. Wickens, W. Wiedenmann, M. Wielers, P. Wienemann, C. Wiglesworth, L. A. M. Wiik-Fuchs, A. Wildauer, F. Wilk, H. G. Wilkens, H. H. Williams, S. Williams, C. Willis, S. Willocq, J. A. Wilson, I. Wingerter-Seez, F. Winklmeier, O. J. Winston, B. T. Winter, M. Wittgen, J. Wittkowski, T. M. H. Wolf, M. W. Wolter, H. Wolters, S. D. Worm, B. K. Wosiek, J. Wotschack, M. J. Woudstra, K. W. Wozniak, M. Wu, M. Wu, S. L. Wu, X. Wu, Y. Wu, T. R. Wyatt, B. M. Wynne, S. Xella, D. Xu, L. Xu, B. Yabsley, S. Yacoob, D. Yamaguchi, Y. Yamaguchi, A. Yamamoto, S. Yamamoto, T. Yamanaka, K. Yamauchi, Y. Yamazaki, Z. Yan, H. Yang, H. Yang, Y. Yang, Z. Yang, W-M. Yao, Y. C. Yap, Y. Yasu, E. Yatsenko, K. H. Yau Wong, J. Ye, S. Ye, I. Yeletskikh, A. L. Yen, E. Yildirim, K. Yorita, R. Yoshida, K. Yoshihara, C. Young, C. J. S. Young, S. Youssef, D. R. Yu, J. Yu, J. M. Yu, J. Yu, L. Yuan, S. P. Y. Yuen, I. Yusuff, B. Zabinski, R. Zaidan, A. M. Zaitsev, N. Zakharchuk, J. Zalieckas, A. Zaman, S. Zambito, L. Zanello, D. Zanzi, C. Zeitnitz, M. Zeman, A. Zemla, J. C. Zeng, Q. Zeng, K. Zengel, O. Zenin, T. Ženiš, D. Zerwas, D. Zhang, F. Zhang, G. Zhang, H. Zhang, J. Zhang, L. Zhang, R. Zhang, R. Zhang, X. Zhang, Z. Zhang, X. Zhao, Y. Zhao, Z. Zhao, A. Zhemchugov, J. Zhong, B. Zhou, C. Zhou, L. Zhou, L. Zhou, M. Zhou, N. Zhou, C. G. Zhu, H. Zhu, J. Zhu, Y. Zhu, X. Zhuang, K. Zhukov, A. Zibell, D. Zieminska, N. I. Zimine, C. Zimmermann, S. Zimmermann, Z. Zinonos, M. Zinser, M. Ziolkowski, L. Živković, G. Zobernig, A. Zoccoli, M. zur Nedden, L. Zwalinski, ATLAS Collaboration

**Affiliations:** 1 Department of Physics, University of Adelaide, Adelaide, Australia; 2 Physics Department, SUNY Albany, Albany, NY USA; 3 Department of Physics, University of Alberta, Edmonton, AB Canada; 4 Department of Physics, Ankara University, Ankara, Turkey; 5 Istanbul Aydin University, Istanbul, Turkey; 6 Division of Physics, TOBB University of Economics and Technology, Ankara, Turkey; 7 LAPP, CNRS/IN2P3 and Université Savoie Mont Blanc, Annecy-le-Vieux, France; 8 High Energy Physics Division, Argonne National Laboratory, Argonne, IL USA; 9 Department of Physics, University of Arizona, Tucson, AZ USA; 10 Department of Physics, The University of Texas at Arlington, Arlington, TX USA; 11 Physics Department, University of Athens, Athens, Greece; 12 Physics Department, National Technical University of Athens, Zografou, Greece; 13 Department of Physics, The University of Texas at Austin, Austin, TX USA; 14 Institute of Physics, Azerbaijan Academy of Sciences, Baku, Azerbaijan; 15 Institut de Física d’Altes Energies (IFAE), The Barcelona Institute of Science and Technology, Barcelona, Spain; 16 Institute of Physics, University of Belgrade, Belgrade, Serbia; 17 Department for Physics and Technology, University of Bergen, Bergen, Norway; 18 Physics Division, Lawrence Berkeley National Laboratory, University of California, Berkeley, CA USA; 19 Department of Physics, Humboldt University, Berlin, Germany; 20 Albert Einstein Center for Fundamental Physics and Laboratory for High Energy Physics, University of Bern, Bern, Switzerland; 21 School of Physics and Astronomy, University of Birmingham, Birmingham, UK; 22 Department of Physics, Bogazici University, Istanbul, Turkey; 23 Department of Physics Engineering, Gaziantep University, Gaziantep, Turkey; 24 Faculty of Engineering and Natural Sciences, Istanbul Bilgi University, Istanbul, Turkey; 25 Faculty of Engineering and Natural Sciences, Bahcesehir University, Istanbul, Turkey; 26 Centro de Investigaciones, Universidad Antonio Narino, Bogota, Colombia; 27 INFN Sezione di Bologna, Bologna, Italy; 28 Dipartimento di Fisica e Astronomia, Università di Bologna, Bologna, Italy; 29 Physikalisches Institut, University of Bonn, Bonn, Germany; 30 Department of Physics, Boston University, Boston, MA USA; 31 Department of Physics, Brandeis University, Waltham, MA USA; 32 Universidade Federal do Rio De Janeiro COPPE/EE/IF, Rio de Janeiro, Brazil; 33 Electrical Circuits Department, Federal University of Juiz de Fora (UFJF), Juiz de Fora, Brazil; 34 Federal University of Sao Joao del Rei (UFSJ), Sao Joao del Rei, Brazil; 35 Instituto de Fisica, Universidade de Sao Paulo, Sao Paulo, Brazil; 36 Physics Department, Brookhaven National Laboratory, Upton, NY USA; 37 Transilvania University of Brasov, Brasov, Romania; 38 National Institute of Physics and Nuclear Engineering, Bucharest, Romania; 39 Physics Department, National Institute for Research and Development of Isotopic and Molecular Technologies, Cluj Napoca, Romania; 40 University Politehnica Bucharest, Bucharest, Romania; 41 West University in Timisoara, Timisoara, Romania; 42 Departamento de Física, Universidad de Buenos Aires, Buenos Aires, Argentina; 43 Cavendish Laboratory, University of Cambridge, Cambridge, UK; 44 Department of Physics, Carleton University, Ottawa, ON Canada; 45 CERN, Geneva, Switzerland; 46 Enrico Fermi Institute, University of Chicago, Chicago, IL USA; 47 Departamento de Física, Pontificia Universidad Católica de Chile, Santiago, Chile; 48 Departamento de Física, Universidad Técnica Federico Santa María, Valparaíso, Chile; 49 Institute of High Energy Physics, Chinese Academy of Sciences, Beijing, China; 50 Department of Physics, Nanjing University, Jiangsu, China; 51 Physics Department, Tsinghua University, Beijing, 100084 China; 52 Laboratoire de Physique Corpusculaire, Clermont Université and Université Blaise Pascal and CNRS/IN2P3, Clermont-Ferrand, France; 53 Nevis Laboratory, Columbia University, Irvington, NY USA; 54 Niels Bohr Institute, University of Copenhagen, Kobenhavn, Denmark; 55 INFN Gruppo Collegato di Cosenza, Laboratori Nazionali di Frascati, Rende, Italy; 56 Dipartimento di Fisica, Università della Calabria, Rende, Italy; 57 Faculty of Physics and Applied Computer Science, AGH University of Science and Technology, Kraków, Poland; 58 Marian Smoluchowski Institute of Physics, Jagiellonian University, Kraków, Poland; 59 Institute of Nuclear Physics, Polish Academy of Sciences, Kraków, Poland; 60 Physics Department, Southern Methodist University, Dallas, TX USA; 61 Physics Department, University of Texas at Dallas, Richardson, TX USA; 62 DESY, Hamburg and Zeuthen, Germany; 63 Lehrstuhl für Experimentelle Physik IV, Technische Universität Dortmund, Dortmund, Germany; 64 Institut für Kern-und Teilchenphysik, Technische Universität Dresden, Dresden, Germany; 65 Department of Physics, Duke University, Durham, NC USA; 66 SUPA-School of Physics and Astronomy, University of Edinburgh, Edinburgh, UK; 67 INFN Laboratori Nazionali di Frascati, Frascati, Italy; 68 Fakultät für Mathematik und Physik, Albert-Ludwigs-Universität, Freiburg, Germany; 69 Section de Physique, Université de Genève, Geneva, Switzerland; 70 INFN Sezione di Genova, Genoa, Italy; 71 Dipartimento di Fisica, Università di Genova, Genoa, Italy; 72 E. Andronikashvili Institute of Physics, Iv. Javakhishvili Tbilisi State University, Tbilisi, Georgia; 73 High Energy Physics Institute, Tbilisi State University, Tbilisi, Georgia; 74 II Physikalisches Institut, Justus-Liebig-Universität Giessen, Giessen, Germany; 75 SUPA-School of Physics and Astronomy, University of Glasgow, Glasgow, UK; 76 II Physikalisches Institut, Georg-August-Universität, Göttingen, Germany; 77 Laboratoire de Physique Subatomique et de Cosmologie, Université Grenoble-Alpes, CNRS/IN2P3, Grenoble, France; 78 Laboratory for Particle Physics and Cosmology, Harvard University, Cambridge, MA USA; 79 Department of Modern Physics, University of Science and Technology of China, Anhui, China; 80 Kirchhoff-Institut für Physik, Ruprecht-Karls-Universität Heidelberg, Heidelberg, Germany; 81 Physikalisches Institut, Ruprecht-Karls-Universität Heidelberg, Heidelberg, Germany; 82 ZITI Institut für technische Informatik, Ruprecht-Karls-Universität Heidelberg, Mannheim, Germany; 83 Faculty of Applied Information Science, Hiroshima Institute of Technology, Hiroshima, Japan; 84 Department of Physics, The Chinese University of Hong Kong, Shatin, NT Hong Kong; 85 Department of Physics, The University of Hong Kong, Pokfulam, Hong Kong; 86 Department of Physics and Institute for Advanced Study, The Hong Kong University of Science and Technology, Clear Water Bay, Kowloon, Hong Kong, China; 87 Department of Physics, Indiana University, Bloomington, IN USA; 88 Institut für Astro- und Teilchenphysik, Leopold-Franzens-Universität, Innsbruck, Austria; 89 University of Iowa, Iowa City, IA USA; 90 Department of Physics and Astronomy, Iowa State University, Ames, IA USA; 91 Joint Institute for Nuclear Research, JINR Dubna, Dubna, Russia; 92 KEK, High Energy Accelerator Research Organization, Tsukuba, Japan; 93 Graduate School of Science, Kobe University, Kobe, Japan; 94 Faculty of Science, Kyoto University, Kyoto, Japan; 95 Kyoto University of Education, Kyoto, Japan; 96 Department of Physics, Kyushu University, Fukuoka, Japan; 97 Instituto de Física La Plata, Universidad Nacional de La Plata and CONICET, La Plata, Argentina; 98 Physics Department, Lancaster University, Lancaster, UK; 99 INFN Sezione di Lecce, Lecce, Italy; 100 Dipartimento di Matematica e Fisica, Università del Salento, Lecce, Italy; 101 Oliver Lodge Laboratory, University of Liverpool, Liverpool, UK; 102 Department of Physics, Jožef Stefan Institute and University of Ljubljana, Ljubljana, Slovenia; 103 School of Physics and Astronomy, Queen Mary University of London, London, UK; 104 Department of Physics, Royal Holloway University of London, Surrey, UK; 105 Department of Physics and Astronomy, University College London, London, UK; 106 Louisiana Tech University, Ruston, LA USA; 107 Laboratoire de Physique Nucléaire et de Hautes Energies, UPMC and Université Paris-Diderot and CNRS/IN2P3, Paris, France; 108 Fysiska institutionen, Lunds universitet, Lund, Sweden; 109 Departamento de Fisica Teorica C-15, Universidad Autonoma de Madrid, Madrid, Spain; 110 Institut für Physik, Universität Mainz, Mainz, Germany; 111 School of Physics and Astronomy, University of Manchester, Manchester, UK; 112 CPPM, Aix-Marseille Université and CNRS/IN2P3, Marseille, France; 113 Department of Physics, University of Massachusetts, Amherst, MA USA; 114 Department of Physics, McGill University, Montreal, QC Canada; 115 School of Physics, University of Melbourne, Victoria, Australia; 116 Department of Physics, The University of Michigan, Ann Arbor, MI USA; 117 Department of Physics and Astronomy, Michigan State University, East Lansing, MI USA; 118 INFN Sezione di Milano, Milan, Italy; 119 Dipartimento di Fisica, Università di Milano, Milan, Italy; 120 B.I. Stepanov Institute of Physics, National Academy of Sciences of Belarus, Minsk, Republic of Belarus; 121 National Scientific and Educational Centre for Particle and High Energy Physics, Minsk, Republic of Belarus; 122 Group of Particle Physics, University of Montreal, Montreal, QC Canada; 123 P.N. Lebedev Physical Institute of the Russian, Academy of Sciences, Moscow, Russia; 124 Institute for Theoretical and Experimental Physics (ITEP), Moscow, Russia; 125 National Research Nuclear University MEPhI, Moscow, Russia; 126 D.V. Skobeltsyn Institute of Nuclear Physics, M.V. Lomonosov Moscow State University, Moscow, Russia; 127 Fakultät für Physik, Ludwig-Maximilians-Universität München, Munich, Germany; 128 Max-Planck-Institut für Physik (Werner-Heisenberg-Institut), Munich, Germany; 129 Nagasaki Institute of Applied Science, Nagasaki, Japan; 130 Graduate School of Science and Kobayashi-Maskawa Institute, Nagoya University, Nagoya, Japan; 131 INFN Sezione di Napoli, Naples, Italy; 132 Dipartimento di Fisica, Università di Napoli, Naples, Italy; 133 Department of Physics and Astronomy, University of New Mexico, Albuquerque, NM USA; 134 Institute for Mathematics Astrophysics and Particle Physics, Radboud University Nijmegen/Nikhef, Nijmegen, The Netherlands; 135 Nikhef National Institute for Subatomic Physics and University of Amsterdam, Amsterdam, The Netherlands; 136 Department of Physics, Northern Illinois University, DeKalb, IL USA; 137 Budker Institute of Nuclear Physics, SB RAS, Novosibirsk, Russia; 138 Department of Physics, New York University, New York, NY USA; 139 Ohio State University, Columbus, OH USA; 140 Faculty of Science, Okayama University, Okayama, Japan; 141 Homer L. Dodge Department of Physics and Astronomy, University of Oklahoma, Norman, OK USA; 142 Department of Physics, Oklahoma State University, Stillwater, OK USA; 143 Palacký University, RCPTM, Olomouc, Czech Republic; 144 Center for High Energy Physics, University of Oregon, Eugene, OR USA; 145 LAL, Univ. Paris-Sud, CNRS/IN2P3, Université Paris-Saclay, Orsay, France; 146 Graduate School of Science, Osaka University, Osaka, Japan; 147 Department of Physics, University of Oslo, Oslo, Norway; 148 Department of Physics, Oxford University, Oxford, UK; 149 INFN Sezione di Pavia, Pavia, Italy; 150 Dipartimento di Fisica, Università di Pavia, Pavia, Italy; 151 Department of Physics, University of Pennsylvania, Philadelphia, PA USA; 152 National Research Centre “Kurchatov Institute” B.P. Konstantinov Petersburg Nuclear Physics Institute, St. Petersburg, Russia; 153 INFN Sezione di Pisa, Pisa, Italy; 154 Dipartimento di Fisica E. Fermi, Università di Pisa, Pisa, Italy; 155 Department of Physics and Astronomy, University of Pittsburgh, Pittsburgh, PA USA; 156 Laboratório de Instrumentação e Física Experimental de Partículas-LIP, Lisbon, Portugal; 157 Faculdade de Ciências, Universidade de Lisboa, Lisbon, Portugal; 158 Department of Physics, University of Coimbra, Coimbra, Portugal; 159 Centro de Física Nuclear da Universidade de Lisboa, Lisbon, Portugal; 160 Departamento de Fisica, Universidade do Minho, Braga, Portugal; 161 Departamento de Fisica Teorica y del Cosmos and CAFPE, Universidad de Granada, Granada, Spain; 162 Dep Fisica and CEFITEC of Faculdade de Ciencias e Tecnologia, Universidade Nova de Lisboa, Caparica, Portugal; 163 Institute of Physics, Academy of Sciences of the Czech Republic, Prague, Czech Republic; 164 Czech Technical University in Prague, Prague, Czech Republic; 165 Faculty of Mathematics and Physics, Charles University in Prague, Prague, Czech Republic; 166 State Research Center Institute for High Energy Physics Protvino, Protvino, Russia; 167 Particle Physics Department, Rutherford Appleton Laboratory, Didcot, UK; 168 INFN Sezione di Roma, Rome, Italy; 169 Dipartimento di Fisica, Sapienza Università di Roma, Rome, Italy; 170 INFN Sezione di Roma Tor Vergata, Rome, Italy; 171 Dipartimento di Fisica, Università di Roma Tor Vergata, Rome, Italy; 172 INFN Sezione di Roma Tre, Rome, Italy; 173 Dipartimento di Matematica e Fisica, Università Roma Tre, Rome, Italy; 174 Faculté des Sciences Ain Chock, Réseau Universitaire de Physique des Hautes Energies-Université Hassan II, Casablanca, Morocco; 175 Centre National de l’Energie des Sciences Techniques Nucleaires, Rabat, Morocco; 176 Faculté des Sciences Semlalia, Université Cadi Ayyad, LPHEA-Marrakech, Marrakesh, Morocco; 177 Faculté des Sciences, Université Mohamed Premier and LPTPM, Oujda, Morocco; 178 Faculté des , Sciences, Université Mohammed V, Rabat, Morocco; 179 DSM/IRFU (Institut de Recherches sur les Lois Fondamentales de l’Univers), CEA Saclay (Commissariat à l’Energie Atomique et aux Energies Alternatives), Gif-sur-Yvette, France; 180 Santa Cruz Institute for Particle Physics, University of California Santa Cruz, Santa Cruz, CA USA; 181 Department of Physics, University of Washington, Seattle, WA USA; 182 School of Physics, Shandong University, Shandong, China; 183 Department of Physics and Astronomy (also affiliated with PKU-CHEP), Shanghai Key Laboratory for Particle Physics and Cosmology, Shanghai Jiao Tong University, Shanghai, China; 184 Department of Physics and Astronomy, University of Sheffield, Sheffield, UK; 185 Department of Physics, Shinshu University, Nagano, Japan; 186 Fachbereich Physik, Universität Siegen, Siegen, Germany; 187 Department of Physics, Simon Fraser University, Burnaby, BC Canada; 188 SLAC National Accelerator Laboratory, Stanford, CA USA; 189 Faculty of Mathematics, Physics and Informatics, Comenius University, Bratislava, Slovakia; 190 Department of Subnuclear Physics, Institute of Experimental Physics of the Slovak Academy of Sciences, Kosice, Slovak Republic; 191 Department of Physics, University of Cape Town, Cape Town, South Africa; 192 Department of Physics, University of Johannesburg, Johannesburg, South Africa; 193 School of Physics, University of the Witwatersrand, Johannesburg, South Africa; 194 Department of Physics, Stockholm University, Stockholm, Sweden; 195 The Oskar Klein Centre, Stockholm, Sweden; 196 Physics Department, Royal Institute of Technology, Stockholm, Sweden; 197 Departments of Physics and Astronomy and Chemistry, Stony Brook University, Stony Brook, NY USA; 198 Department of Physics and Astronomy, University of Sussex, Brighton, UK; 199 School of Physics, University of Sydney, Sydney, Australia; 200 Institute of Physics, Academia Sinica, Taipei, Taiwan; 201 Department of Physics, Technion: Israel Institute of Technology, Haifa, Israel; 202 Raymond and Beverly Sackler School of Physics and Astronomy, Tel Aviv University, Tel Aviv, Israel; 203 Department of Physics, Aristotle University of Thessaloniki, Thessaloniki, Greece; 204 International Center for Elementary Particle Physics and Department of Physics, The University of Tokyo, Tokyo, Japan; 205 Graduate School of Science and Technology, Tokyo Metropolitan University, Tokyo, Japan; 206 Department of Physics, Tokyo Institute of Technology, Tokyo, Japan; 207 Tomsk State University, Tomsk, Russia Russia; 208 Department of Physics, University of Toronto, Toronto, ON Canada; 209 INFN-TIFPA, Povo, Italy; 210 University of Trento, Trento, Italy Italy; 211 TRIUMF, Vancouver, BC Canada; 212 Department of Physics and Astronomy, York University, Toronto, ON Canada; 213 Faculty of Pure and Applied Sciences, Center for Integrated Research in Fundamental Science and Engineering, University of Tsukuba, Tsukuba, Japan; 214 Department of Physics and Astronomy, Tufts University, Medford, MA USA; 215 Department of Physics and Astronomy, University of California Irvine, Irvine, CA USA; 216 INFN Gruppo Collegato di Udine, Sezione di Trieste, Udine, Italy; 217 ICTP, Trieste, Italy; 218 Dipartimento di Chimica, Fisica e Ambiente, Università di Udine, Udine, Italy; 219 Department of Physics and Astronomy, University of Uppsala, Uppsala, Sweden; 220 Department of Physics, University of Illinois, Urbana, IL USA; 221 Instituto de Fisica Corpuscular (IFIC) and Departamento de Fisica Atomica, Molecular y Nuclear and Departamento de Ingeniería Electrónica and Instituto de Microelectrónica de Barcelona (IMB-CNM), University of Valencia and CSIC, Valencia, Spain; 222 Department of Physics, University of British Columbia, Vancouver, BC Canada; 223 Department of Physics and Astronomy, University of Victoria, Victoria, BC Canada; 224 Department of Physics, University of Warwick, Coventry, UK; 225 Waseda University, Tokyo, Japan; 226 Department of Particle Physics, The Weizmann Institute of Science, Rehovot, Israel; 227 Department of Physics, University of Wisconsin, Madison, WI USA; 228 Fakultät für Physik und Astronomie, Julius-Maximilians-Universität, Würzburg, Germany; 229 Fakultät für Mathematik und Naturwissenschaften, Fachgruppe Physik, Bergische Universität Wuppertal, Wuppertal, Germany; 230 Department of Physics, Yale University, New Haven, CT USA; 231 Yerevan Physics Institute, Yerevan, Armenia; 232 Centre de Calcul de l’Institut National de Physique Nucléaire et de Physique des Particules (IN2P3), Villeurbanne, France; 233 CERN, 1211 Geneva 23, Switzerland

## Abstract

This paper presents a dedicated search for exotic decays of the Higgs boson to a pair of new spin-zero particles, $H \rightarrow aa$, where the particle *a* decays to *b*-quarks and has a mass in the range of 20–60 GeV. The search is performed in events where the Higgs boson is produced in association with a $W$ boson, giving rise to a signature of a lepton (electron or muon), missing transverse momentum, and multiple jets from *b*-quark decays. The analysis is based on the full dataset of *pp* collisions at $\sqrt{s} = 13\,\mathrm{{TeV}}$ recorded in 2015 by the ATLAS detector at the CERN Large Hadron Collider, corresponding to an integrated luminosity of 3.2 $\text{ fb }^{-1}$. No significant excess of events above the Standard Model prediction is observed, and a $95\,\%$ confidence-level upper limit is derived for the product of the production cross section for $pp \rightarrow WH$ times the branching ratio for the decay $H \rightarrow aa \rightarrow 4b$. The upper limit ranges from 6.2 pb for an *a*-boson mass $m_a = 20\,\mathrm{{GeV}}$ to 1.5 pb for $m_a = 60\,\mathrm{{GeV}}$.

## Introduction

Following the discovery of the Higgs boson by the ATLAS and CMS Collaborations [1, 2] at the Large Hadron Collider (LHC), a comprehensive programme of measurements of the properties of this particle is underway. These measurements could uncover deviations from expected Standard Model (SM) branching ratios or allow for the possibility of decays into non-SM particles. Existing measurements constrain the non-SM or “exotic” branching ratio of the Higgs boson decays to less than approximately $30\,\%$ at $95\,\%$ confidence level (CL) [3–5]. Exotic decays are predicted by many theories of physics beyond the SM [6], including those with an extended Higgs sector such as the Next-to-Minimal Supersymmetric Standard Model (NMSSM) [7–11], several models of dark matter [12–16], models with a first-order electroweak phase transition [17, 18], and theories with neutral naturalness [19–21].

One of the simplest possibilities is that the Higgs boson decays to a pair of new spin-zero particles, *a*, which in turn decay to a pair of SM particles, mainly fermions [6].^1^ These kinds of models have been used to explain the recent observations of a gamma-ray excess from the galactic centre by the Fermi Large Area Telescope (FermiLAT) [22, 23]. Several searches have been performed for $H\rightarrow aa$. The D0 and ATLAS Collaborations have searched for a signal of $H\rightarrow aa \rightarrow 2\mu 2\tau$ in the *a*-boson mass ranges $3.7\,\mathrm{{GeV}} \le m_a \le 19\,\mathrm{{GeV}}$ and $3.7\,\mathrm{{GeV}} \le m_a \le 50\,\mathrm{{GeV}}$, respectively [24, 25]. The D0 and CMS Collaborations have searched for the signature $H \rightarrow aa \rightarrow 4\mu$ in the range $2 m_{\mu } \le m_a \le 2 m_{\tau }$ [24, 26]. In this analysis, the *a*-boson is assumed to have a negligibly small lifetime. Several other searches have been performed by the ATLAS, CMS and LHCb Collaborations for signatures that may correspond to a long-lived *a*-boson: displaced decays of jets or displaced decays of collimated leptons [27–32].

The result presented in this paper covers an unexplored decay mode in searches for $H \rightarrow aa$ by considering $a\rightarrow bb$. The *a*-boson can be either a scalar or a pseudoscalar under parity transformations, since the decay mode considered in this search is not sensitive to the difference in coupling. An example of a model with predominant $a\rightarrow bb$ decays is one where the new scalar mixes with the SM Higgs boson and inherits its Yukawa couplings [6]. This search focuses on the $pp \rightarrow WH$ process, with $W\rightarrow \ell \nu$ ($\ell =e,\mu$) and $H \rightarrow 2a \rightarrow 4b$ in the range $20\,\mathrm{{GeV}}<m_a<60\,\mathrm{{GeV}}$. The resulting signature has a single lepton accompanied by a high multiplicity of jets originating from a bottom quark (*b*-jets). Since the *b*-jets are produced from the decay of the Higgs boson, they tend to have low transverse momentum ($p_{\text {T}}$) compared to $m_H$ and can be overlapping, especially for light *a*-bosons. Events with an electron or muon, including those produced via leptonically decaying $\tau$-leptons, are considered. The *WH* process is chosen for this search because the charged lepton in the final state provides a powerful handle to efficiently trigger and identify these events against the more ubiquitous background process of strong production of four *b*-jets. Several kinematic variables, including the reconstructed masses in the decay $H\rightarrow 2a \rightarrow 4b$, are used to identify signal events. The background estimation techniques, systematic uncertainties and statistical treatment closely follow those used in other ATLAS searches with similar signatures [33–36].

## ATLAS detector

The ATLAS detector [37] covers nearly the entire solid angle^2^ around the collision point. It consists of an inner tracking detector surrounded by a thin superconducting solenoid magnet producing a 2 T axial magnetic field, electromagnetic and hadronic calorimeters, and an external muon spectrometer incorporating three large toroid magnet assemblies. The inner detector consists of a high-granularity silicon pixel detector, including the newly installed insertable B-layer [38], and a silicon microstrip tracker, together providing precision tracking in the pseudorapidity range $|\eta |<2.5$, complemented by a transition radiation tracker providing tracking and electron identification information for $|\eta |<2.0$. The electromagnetic (EM) sampling calorimeter uses lead as the absorber material and liquid argon (LAr) as the active medium, and is divided into barrel ($|\eta |<1.475$) and end-cap ($1.375<|\eta |<3.2$) regions. Hadron calorimetry is also based on the sampling technique, with either scintillator tiles or LAr as the active medium, and with steel, copper, or tungsten as the absorber material. The scintillator tile calorimeter is divided into barrel ($|\eta |<1.0$) and end-cap ($0.8<|\eta |<1.7$) regions, and the LAr hadronic calorimeter includes an end-cap ($1.5<|\eta |<3.2$) and a forward ($3.1<|\eta |<4.9$) region. The muon spectrometer measures the deflection of muons with $|\eta |<2.7$ using multiple layers of high-precision tracking chambers in a toroidal field of approximately 0.5 T and 1 T in the central and end-cap regions of ATLAS, respectively. The muon spectrometer is also instrumented with separate trigger chambers covering $|\eta |<2.4$. A two-level trigger system, consisting of a custom-hardware level followed by a software-based level, is used to reduce the event rate to a maximum of around 1 kHz for offline storage [39].

## Event samples and object selection

The search presented in this paper is based on the proton–proton (*pp*) collision dataset collected by the ATLAS detector at the LHC at $\sqrt{s}=13 \,\mathrm{{TeV}}$ with 25 ns bunch spacing during 2015. The full dataset corresponds to an integrated luminosity of $3.2 \,{\text{ fb }^{-1}}$. The data for this search were collected using the single-electron or single-muon triggers with the lowest transverse momentum thresholds available [39].

Electron candidates are reconstructed by associating an inner-detector track with an isolated energy deposit in the EM calorimeter [40, 41]. Candidates are identified using the tight quality criteria and are required to have $p_{\text {T}} >25\,\mathrm{{GeV}}$ and $|\eta |<2.47$, excluding the transition region between the barrel and end-cap EM calorimeters, $1.37<|\eta |<1.52$. Muon candidates are reconstructed by combining matching tracks in the inner detector and the muon spectrometer [42], and are required to satisfy the medium quality criteria and to have $p_{\text {T}} >25\,\mathrm{{GeV}}$ and $|\eta |<2.4$. Events are required to have exactly one reconstructed electron or muon that is matched within a cone of size $\Delta R \equiv \sqrt{(\Delta \eta )^2 + (\Delta \phi )^2} = 0.15$ to the lepton candidate reconstructed by the trigger algorithms.

In order to distinguish leptons produced in the decays of $W$ bosons from those produced in the decays of heavy-flavour hadrons, all lepton candidates are required to be consistent with originating from the primary interaction vertex, chosen as the vertex with the highest sum of the $p_{\text {T}} ^2$ of its associated tracks. Furthermore, since lepton candidates arising from background sources, such as decays of hadrons, are typically embedded in jets, all lepton candidates are required to be isolated from other particles in the event. This is achieved by imposing a maximal allowed value on the energy deposited in the calorimeter and/or the momentum of inner-detector tracks within a cone of $\Delta R = 0.2$ around the direction of the lepton candidate’s momentum. The isolation criteria, dependent on $p_{\text {T}}$ and $\eta$, are applied to produce a nominal efficiency of at least $90\,\%$ for electrons and muons from $Z\rightarrow \ell ^+\ell ^-$ decays after all other identification criteria are applied [42].

Jets are reconstructed from clusters [43] of energy in the calorimeters using the anti-$k_{t}$ clustering algorithm [44, 45] with radius parameter $R=0.4$. Jets are required to have $p_{\text {T}} > 20\,\mathrm{{GeV}}$ and $|\eta |<2.5$, and they are calibrated using energy- and $\eta$-dependent corrections. A track-based veto is used to suppress contributions from jets arising from additional *pp* interactions (pile-up) [46]. Jets consistent with the hadronisation of a *b*-quark are identified using information from track impact parameters and secondary vertices, which are combined in a multivariate discriminant [47]. The efficiency to identify *b*-quark jets (*b*-tagging) is approximately $77\,\%$ for a factor of 126 in rejection against light-quark and gluon jets, about 5 against jets originating from *c*-quarks, and about 10 against hadronically decaying $\tau$-leptons, as determined in a simulated sample of top-quark pair ($t\bar{t}$) events [47–49]. Jets tagged by this multivariate discriminant, independently of the flavour of the quark that initiated it, are called *b*-tagged jets throughout the text, while the term *b*-jet is reserved for those jets originating from *b*-quark decays, as determined from simulation.

Jets are required to be separated from the lepton candidates by $\Delta R$ larger than 0.2 or 0.4 for electrons or muons, respectively. Electrons separated from the nearest jet by $0.2<\Delta R < 0.4$ are considered part of the jet and not a lepton candidate. The transverse momentum imbalance $\vec {E}_{\text {T}}^{\text {miss}}$, the magnitude of which ($E_{\text {T}}^{\text {miss}}$) is commonly referred to as missing transverse momentum, is defined as the negative vector sum of the transverse momenta of calibrated selected objects, such as electrons, muons and jets. The transverse momenta of charged-particle tracks compatible with the primary vertex and not matched to any of those objects are also included in the negative vector sum [50, 51].

## Signal and background modelling

Simulated event samples are used to study the characteristics of the signal and to calculate its acceptance, and are also used for most of the SM background estimation. Signal samples of associated Higgs boson production with a $W$ boson, $pp \rightarrow W H$, are generated with Powheg-Box v2–r3033 [52–55] using the CT10 parton distribution functions (PDFs) [56] at next-to-leading order (NLO). A Higgs boson mass of $m_H = 125\,\mathrm{{GeV}}$ is assumed and the sample is normalised to the next-to-next-to-leading-order (NNLO) cross section recommended by the Higgs cross-section working group $\sigma _\mathrm{SM}(WH) = 1.37$ pb [57]. The Higgs boson decay to two spin-zero *a*-bosons and the subsequent decay of each *a*-boson to a pair of *b*-quarks are simulated with Pythia  v8.186 [58]. The *a*-boson decay is done in the narrow-width approximation and the coupling to the *b*-quarks is assumed to be that of a pseudoscalar. However, since the polarisation of the quarks is not observable, this search is insensitive to the specific parity hypothesis. Pythia  v8.186 is used for the showering, hadronisation, and underlying-event (UE) simulation with the A14 set of tuned parameters (tune) [59]. The mass of the *a*-boson is varied for different signal hypotheses in the range $20\,\mathrm{{GeV}} \le m_a \le 60\,\mathrm{{GeV}}$, in $10\,\mathrm{{GeV}}$ mass steps. Different branching-ratio hypotheses are obtained by scaling the signal sample normalisation.

Samples of $t\bar{t}$ are also produced using the NLO Powheg-Box v2–r3026 generator with the CT10 PDFs. A top-quark mass ($m_t$) of $172.5\,\mathrm {GeV}$ is assumed. The Powheg-Box model parameter $h_\mathrm{damp}$, which controls matrix element to parton shower (PS) matching and effectively regulates the high-$p_{\text {T}}$ radiation, is set to $h_\mathrm{damp} = m_t$. This setting was found to best describe the $t\bar{t}$-system $p_{\text {T}}$ at $\sqrt{s} = 7\mathrm {TeV}$ [60]. The baseline $t\bar{t}$ sample is interfaced to Pythia  v6.428 [61] with the Perugia 2012 tune [62]. Alternative $t\bar{t}$ samples are generated using Powheg-Box v2–r3026 interfaced to Herwig++ v2.7 [63] or MadGraph5_aMC@NLO [64] interfaced to Herwig++. The effects of initial- and final-state radiation (ISR/FSR) are explored using two alternative Powheg-Box  v2–r3026+Pythia  v6.428 samples. The first has $h_\mathrm{damp}$ set to $2 m_t$, the renormalisation and factorisation scales set to half the nominal value and uses the Perugia 2012 radHi UE tune, giving more radiation. The second sample uses the Perugia 2012 radLo UE tune, has $h_\mathrm{damp}=m_t$ and has the renormalisation and factorisation scales set to twice the nominal value, giving less radiation [65]. The $t\bar{t}$ samples are normalised to the NNLO theoretical cross section of $832^{+46}_{-51}$ pb, obtained with Top++ v2.0 [66–72].

The simulated $t\bar{t}$ events are categorised depending on the parton-level flavour content of additional particle jets^3^ not originating from the decay of the $t\bar{t}$ system. Events containing at least one additional particle jet matched to a *b*-hadron are labelled as $t\bar{t}$. Events containing at least one additional particle jet matched to a *c*-hadron and no *b*-hadron are labelled as $b\bar{b}$. The $t\bar{t}$ and $b\bar{b}$ categories are generically referred to as $t\bar{t}$+HF events (with HF standing for “heavy flavour”). Remaining events are labelled $t\bar{t}$+light-jets (referred to as $t\bar{t}$+light) and also include events with no additional particle jets.

The associated heavy-flavour jets in $t\bar{t}$+HF are modelled in Powheg-Box+Pythia via the PS evolution and are simulated with a five-flavour scheme. The $t\bar{t}$ modelling is improved by reweighting the top-quark $p_{\text {T}}$, $t\bar{t}$-system $p_{\text {T}}$, and kinematic properties of the associated particle jets not originating from the top-quark decay [33] to agree with a $t\bar{t}$ sample generated at NLO with Sherpa+OpenLoops [73, 74]. This Sherpa+OpenLoops sample is simulated with the four-flavour scheme (4FS) using Sherpa v2.1.1 [73] and the CT10 PDF set.

Samples of single-top-quark backgrounds corresponding to the *Wt* and *s*-channel production mechanisms are generated with Powheg-Box v2–r2819 [75, 76] using the CT10 PDF set. Overlaps between the $t\bar{t}$ and *Wt* final states are handled using the “diagram removal” scheme [77]. Samples of *t*-channel single-top-quark events are generated using the Powheg-Box [78] NLO generator that uses the 4FS. The single-top-quark samples are normalised to the approximate NNLO theoretical cross sections [79–81]. The parton shower, hadronisation and underlying event are modelled using either Pythia v6.428 with the Perugia 2012 tune or Herwig ++ v2.7 with the UE-EE-5 [82] tune.

Samples of *W* / *Z*+jets events are generated with the Sherpa v2.1.1 generator. The matrix-element calculation is performed up to two partons at NLO and up to four partons at leading order (LO) using Comix [83] and OpenLoops [74] and uses the CT10 PDFs. Both the *W*+jets and *Z*+jets samples are normalised to their respective inclusive NNLO theoretical cross section calculated with FEWZ [84].

Samples of diboson production *WW* / *WZ* / *ZZ*+jets events are generated with the NLO generator Sherpa v2.1.1. Samples of $t\bar{t} + \gamma /W/Z$ events, including $t\bar{t} + WW$, are generated with up to two additional partons using MadGraph5_aMC@NLO and interfaced to Pythia  v8.186. Samples of $t\bar{t} + H$ events are generated using MadGraph5_aMC@NLO and interfaced to Herwig ++ v2.7.

The main signal and background samples use the EvtGen v1.2.0 [85] program to simulate the decay of heavy-flavour hadrons, except for those generated with Sherpa. All are then processed with the full simulation of the ATLAS detector [86] based on GEANT4 [87]. The alternative $t\bar{t}$ samples used to estimate systematic uncertainties are based on a fast simulation of the calorimeter response [88]. Events are generated with pile-up that is simulated with Pythia  v8.186 [58] and are reweighted so that the distribution of the multiplicity of pile-up interactions matches the distribution observed in the data. Simulated event samples are processed using the same reconstruction algorithms and analysis chain as the data.

As described in Sect. 5, backgrounds are estimated by fitting predictions derived from simulation to data in several background-enriched samples. The only background prediction not derived from simulation is the multijet background, which contributes to the selected data sample when a jet is mis-reconstructed as a lepton and satisfies the identification criteria. In the electron channel, it consists of non-prompt electrons from heavy-flavour decays, from unidentified photon conversions or from jets with a high fraction of energy deposited in the EM calorimeter. In the muon channel, it consists of heavy-flavour decays and in-flight decays of light mesons.

The multijet background contribution is evaluated from data using the “matrix method” [34, 89, 90], which uses differences between the isolation properties of background (fake/non-prompt) leptons and signal (prompt) leptons from *W* boson decays. The estimate uses a sample enriched in multijet background events obtained by applying the full event selection except for loosening the lepton isolation requirement. Each event with a lepton candidate that satisfies at least the loosened isolation requirement is scaled by a weight that depends on whether this lepton candidate also satisfies the tighter isolation requirement. The weights are determined from the efficiencies for fake/non-prompt and prompt leptons satisfying the loosened isolation requirement to also satisfy the tighter one [90]. These efficiencies are measured in data control samples enriched in either fake/non-prompt leptons, mostly multijet events, or prompt leptons, mostly $Z\rightarrow \ell ^+\ell ^-$ events. The shape of each multijet background distribution is derived by applying the same method to the sample obtained with an identical selection as described in Sect. 5, but lowering the *b*-tagged-jet multiplicity requirement to two. This strategy reduces the statistical uncertainty of the multijet background estimate, improving the stability of the fitting method described in Sect. 5.2.

**Table 1:** List of variables used in the three signal regions as inputs to the BDT multivariate discriminant and used in the five control regions. The variables are described in the text

Region	$m_{bbb}$	$m_{bbbb}$	$\Delta m_\mathrm{min}^{bb}$	$H_\mathrm{T}$	$p_{\text {T}} ^W$	$\Delta R_\mathrm{av}^{bb}$	$\Delta R_\mathrm{min}^{\ell b}$	$m_{bbj}$	$m_\mathrm{T2}$
Signal									
(3j, 3b)	$\checkmark$			$\checkmark$	$\checkmark$	$\checkmark$	$\checkmark$		
(4j, 3b)	$\checkmark$			$\checkmark$	$\checkmark$	$\checkmark$		$\checkmark$	
(4j, 4b)		$\checkmark$	$\checkmark$	$\checkmark$		$\checkmark$			$\checkmark$
Control				$\checkmark$					

## Analysis strategy

The $H \rightarrow 2a \rightarrow 4b$ decay chain is expected to have multiple *b*-tagged jets, often three or four, satisfying the object selection. The dominant background arises from $t\bar{t}$ events. Preselected events are required to have exactly one electron or muon and at least three jets, of which at least two must be *b*-tagged. Events are required to satisfy $E_{\text {T}}^{\text {miss}} >25\,\mathrm{{GeV}}$ and the transverse mass^4^ must fulfil $m_{\mathrm T}^W>50\,\mathrm{{GeV}}$, in order to be consistent with $W$ boson decays. Events are categorised into eight channels depending on the number of jets (3, 4 and $\ge$5) and the number of *b*-tagged jets (2, 3 and $\ge$4). These analysis channels are referred to as (*n*j, *m*b) indicating *n* selected jets including *m* *b*-tagged jets.

The categories most sensitive to the $H \rightarrow 2a \rightarrow 4b$ decay chain are (3j, 3b), (4j, 3b) and (4j, 4b). In these channels, background $t\bar{t}$ events can only satisfy the selection criteria if accompanied by additional *b*-tagged jets. In the case of (3j, 3b) or (4j, 3b), the main sources of $t\bar{t}$ background are events with jets mis-identified as *b*-jets, particularly from $W \rightarrow cs$ decays, where the *c*-jet is mis-identified, and from $W \rightarrow \tau \nu$, where the $\tau$-lepton decays hadronically and is likewise mis-identified. In the case of (4j, 4b), the $t\bar{t}$ background includes more events with genuine *b*-quarks from gluon splitting to $b\bar{b}$ pairs. The main purpose of the five other jet and *b*-tagged-jet multiplicity channels is to constrain the $t\bar{t}$+jets background prediction and the related systematic uncertainties (see Sect. 6) through a profile likelihood fit to data (see Sect. 5.2).

The $t\bar{t}$+light background is dominant in the sample of events with exactly two or three *b*-tagged jets. The background processes $b\bar{b}$ and $t\bar{t}$ become more important as the jet and *b*-tagged-jet multiplicities increase. In particular, the $t\bar{t}$ background dominates for events with $\ge$5 jets and $\ge$4 *b*-tagged jets.

### Signal and background discrimination

In order to improve the sensitivity of the search, several kinematic variables are identified to distinguish between signal and background, and are combined into a boosted decision tree (BDT) multivariate discriminant [91] that uses the AdaBoost algorithm [92]. The BDT is trained to discriminate between signal events with an *a*-boson mass of 60 GeV and $t\bar{t}$ events. As described below, the variables chosen as input for the BDT do not depend strongly on the value of $m_a$ and provide excellent separation between signal and background, so training each mass hypothesis separately with these variables would only slightly improve the sensitivity of the search. The training is performed separately for each of the channels (3j, 3b), (4j, 3b) and (4j, 4b) since the signal and background kinematics differ between them.

Signal events are characterised by the presence of a resonance resulting from the Higgs boson decay $H \rightarrow 2a \rightarrow 4b$. Two variables are used to reconstruct particles from the signal decay chain. The first is the reconstructed invariant mass of the *b*-tagged jets, $m_{bbb}$ or $m_{bbbb}$, defined for events with three or four *b*-tagged jets respectively, which peaks around the Higgs boson mass for signal events. In the case of three *b*-tagged jets, the peak is due to events where two *b*-quarks are merged in a single jet or one of the *b*-quarks is very soft in an asymmetric decay and has a small impact on the kinematics. The second discriminating variable for events with four *b*-tagged jets is the minimum difference between the invariant masses of *bb* pairs ($\Delta m_\mathrm{min}^{bb}$). For signal events, two pairs of *b*-quarks originate from a pair of *a*-bosons, so for the correct jet pairing, $m_{bb} \approx m_a$, and the difference between the invariant masses of the *bb* pairs is smaller for signal than for $t\bar{t}$ background events.

**Fig. 1:**
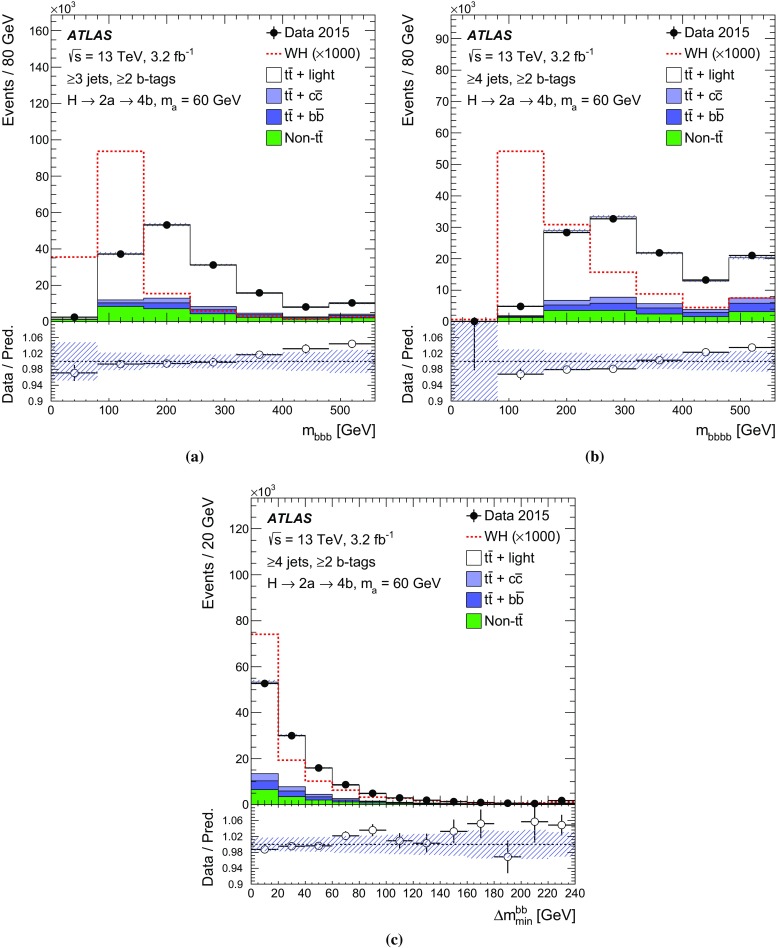
Comparison of data with the SM background predictions for the distributions of **a** $m_{bbb}$, **b** $m_{bbbb}$ and **c** $\Delta m_\mathrm{min}^{bb}$ in the sample that is inclusive in number of jets and *b*-tagged jets. Distributions for the signal model (*WH*, $H\rightarrow 2a \rightarrow 4b$), with $m_a=$ 60 GeV, normalised to the SM $pp \rightarrow WH$ cross section, assuming BR$(H\rightarrow aa)$ $\times$ BR$(a \rightarrow bb)^2 = 1$ and scaled by a factor of 1000, are overlaid. The *hashed area* represents the total uncertainty in the background. Comparisons use events with $\ge$3 jets, except when at least four jets are necessary to define the variable, in which case events with $\ge$4 jets are used. The last bin contains the overflow. Markers are not drawn if they are outside the y-axis range

**Fig. 2:**
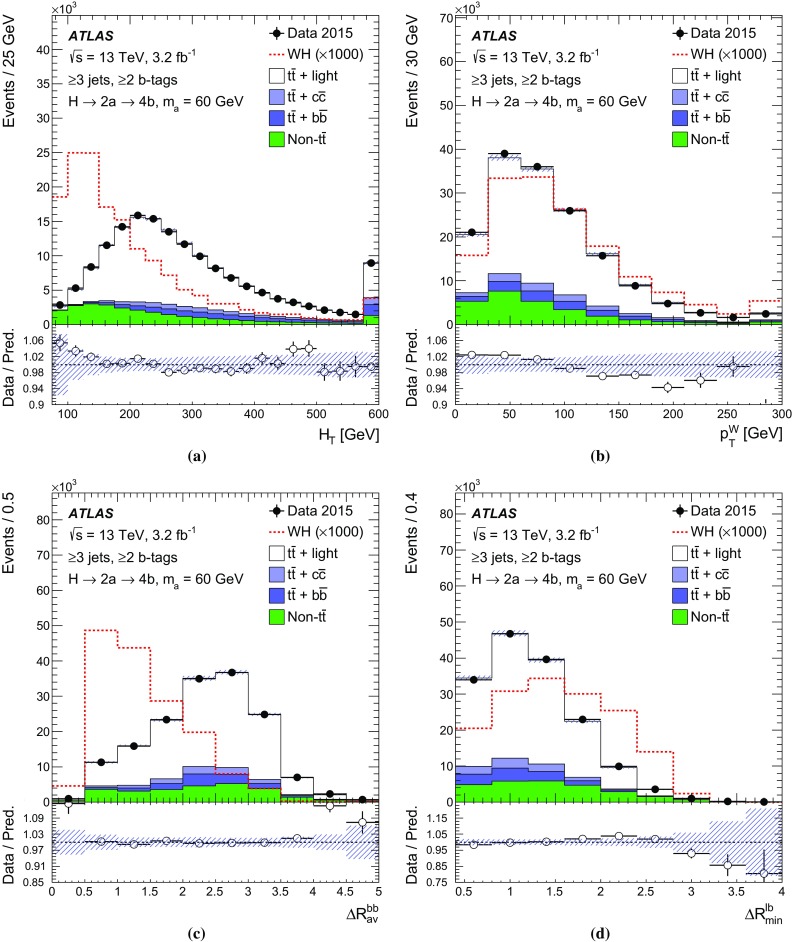
Comparison of data with the SM background predictions for the distributions of **a** $H_\mathrm{T}$, **b** $p_{\text {T}} ^W$, **c** $\Delta R_\mathrm{av}^{bb}$ and **d** $\Delta R_\mathrm{min}^{\ell b}$ in the sample that is inclusive in number of jets and *b*-tagged jets. Distributions for the signal model (*WH*, $H\rightarrow 2a \rightarrow 4b$), with $m_a=60\,\mathrm{{GeV}}$, normalised to the SM $pp \rightarrow WH$ cross section, assuming $\mathrm {BR}(H\rightarrow aa) \times \mathrm {BR}(a \rightarrow bb)^2 = 1$ and scaled by a factor of 1000, are overlaid. The *hashed area* represents the total uncertainty in the background. The last bin contains the overflow

**Fig. 3:**
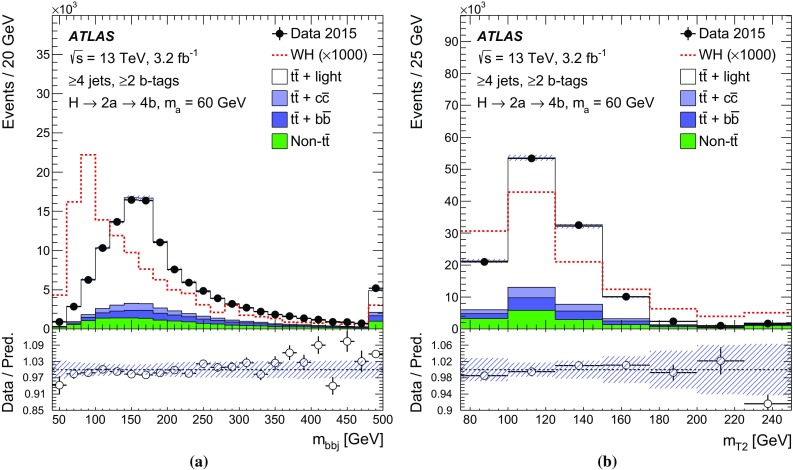
Comparison of data with the SM background predictions for the distributions of **a** $m_{bbj}$ and **b** $m_\mathrm{T2}$ in the sample that is inclusive in number of jets and *b*-tagged jets. Distributions for the signal model (*WH*, $H\rightarrow 2a \rightarrow 4b$), with $m_a=60\,\mathrm{{GeV}}$, normalised to the SM $pp \rightarrow WH$ cross section, assuming $\mathrm {BR}(H\rightarrow aa) \times \mathrm {BR}(a \rightarrow bb)^2 = 1$ and scaled by a factor of 1000, are overlaid. The *hashed area* represents the total uncertainty in the background. The last bin contains the overflow

Additional kinematic variables exhibit differences between signal and background. The $H_\mathrm{T}$ variable, defined as the scalar sum of $p_{\text {T}}$ for all jets in the event, is a measure of the total hadronic energy in the event, which is typically larger for $t\bar{t}$ than for *WH* events. The transverse momentum of the *W* boson, $p_{\text {T}} ^W$, constructed from the vector sum of the $\vec {E}_{\text {T}}^{\text {miss}}$ and the lepton $\vec {p}_{\text {T}}$, is slightly higher for signal *WH* events, where the *W* boson recoils against the Higgs boson, than for background $t\bar{t}$ events. Another variable used is the average angular separation between all pairs of *b*-tagged jets, referred to as $\Delta R_\mathrm{av}^{bb}$. For background $t\bar{t}$ events, the *b*-tagged jets originate from the decays of the two top quarks and tend to be spatially more separated than for the signal. A related variable is the minimum $\Delta R$ separation between any *b*-tagged jet and the lepton, $\Delta R_\mathrm{min}^{\ell b}$. In $t\bar{t}$ background events, the lepton is typically closer to a *b*-tagged jet than in signal events, since the lepton and the nearest *b*-tagged jet often originate from the same top-quark decay. In the case of the signal, the Higgs boson and hence the *b*-jets recoil against the *W* boson, which the lepton comes from.

Finally, two variables are used to identify particles from the dominant $t\bar{t}$ background decay chain. The first variable is used in the (4j, 3b) channel to distinguish between $t\bar{t}$ events with two *b*-tagged jets from the top-quark decays and $t\bar{t}$ events with a third *b*-tagged jet from a mis-identified charm or light jet from the hadronically decaying $W$ boson. The invariant mass of two *b*-tagged jets, selected as the pair with the smallest $\Delta R$ separation, and the non-*b*-tagged jet, $m_{bbj}$, reconstructs the hadronically decaying top quark, peaking around the top-quark mass for these background events. The second variable, used in the (4j, 4b) channel, is a variant of the $m_\mathrm{T2}$ observable, defined as the minimum “mother” particle mass compatible with all the transverse momenta and mass-shell constraints [93], that identifies events with several invisible particles. In the case of the $t\bar{t}$ background events, in addition to the $E_{\text {T}}^{\text {miss}}$ from the neutrino from a leptonic *W* boson decay, invisible particles may arise from a $\tau$-lepton decay or from a lost jet from a *W* boson. In these cases, the $m_\mathrm{T2}$ has an endpoint at the top-quark mass, which is not the case for the signal.

**Fig. 4:**
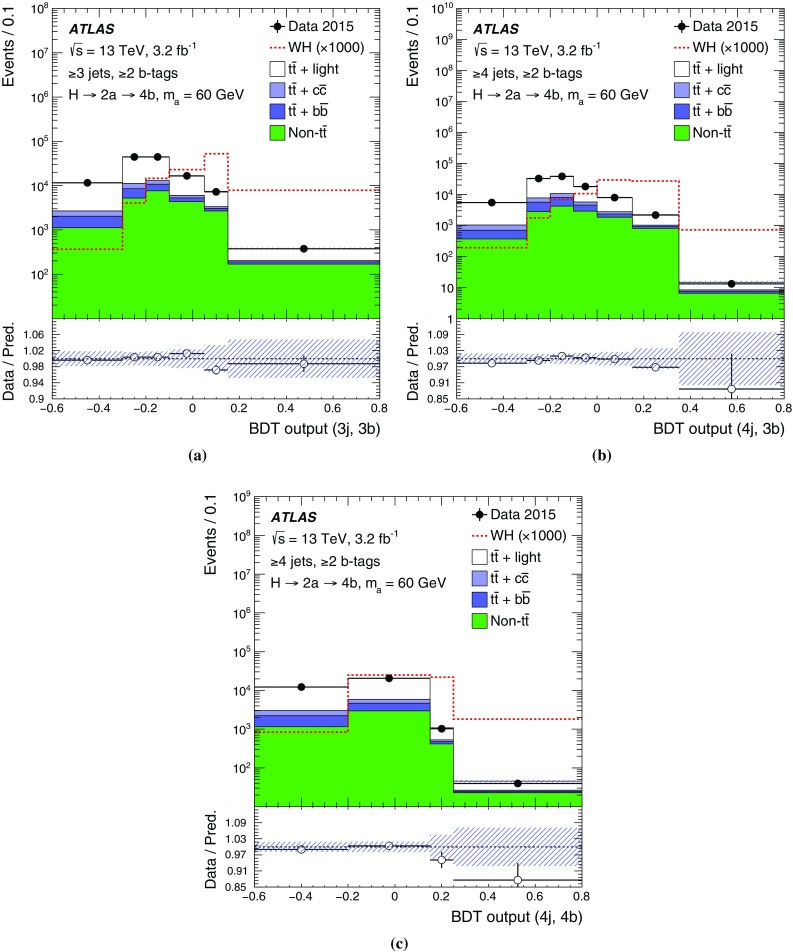
Comparison of data with the SM background predictions for the distributions of **a** BDT (3j, 3b), **b** BDT (4j, 3b), and **c** BDT (4j, 4b) in the sample that is inclusive in number of jets and *b*-tagged jets. Distributions for the signal model (*WH*, $H\rightarrow 2a \rightarrow 4b$), with $m_a=$ 60 GeV, normalised to the SM $pp \rightarrow WH$ cross section, assuming BR$(H\rightarrow aa)$ $\times$ BR$(a \rightarrow bb)^2 = 1$ and scaled by a factor of 1000, are overlaid. The hashed area represents the total uncertainty in the background. Comparisons use events with $\ge$3 jets, except when at least four jets are necessary to define the BDT discriminant, in which case events with $\ge$4 jets are used. The BDT output is determined in the range $[-1,1]$. The first and last bin contain the underflow and overflow, respectively

Table 1 indicates which variables are used to train each of the three BDT discriminants for the (3j, 3b), (4j, 3b), and (4j, 4b) categories. Figures 1, 2 and 3 show the expected distributions of the kinematical variables obtained after using the statistical procedure and the systematic uncertainties described in Sects. 5.2 and 6, respectively. These variables are used in the BDT discriminants for signal and background for all events that satisfy the event selection criteria, and are shown in Figs. 1, 2 and 3 inclusively in number of jets and *b*-tagged jets. The distributions are dominated by events with the minimum number of *b*-tagged jets. In this comparison, the jets in each event are ordered by value of the *b*-tagging discriminant and those with the highest score are used to calculate the input variables of the BDT, even if they do not satisfy the *b*-tagging criteria used in this analysis. The distributions are similar to those obtained in each analysis channel and indicate that each variable individually has some signal and background discrimination power. The tail in the $m_{bbbb}$ distribution for signal events, shown in Fig. 1, is mainly formed by events with jets mis-associated to the *a*-boson decay. The tail is greatly reduced in the signal regions with the tighter requirement on the number of *b*-tagged jets. Figure 4 shows the BDT discriminant for signal and background events that satisfy the event selection criteria inclusively in number of jets and *b*-tagged jets. These distributions are used to validate the BDT modelling in background-enriched samples with kinematic properties that are similar to those in the signal regions.

### Fitting procedure

The distributions of the final discriminants in the eight analysis channels considered are combined to test the presence of a signal. The BDT discriminant, described in Sect. 5.1, is used for the channels enriched with signal, (3j, 3b), (4j, 3b) and (4j, 4b), while the $H_{\text {T}}$ distribution is used in the five control channels. The statistical analysis is based on a binned likelihood function constructed as a product of Poisson probability terms over all bins considered in the search.

The likelihood function, *L*, depends on the parameter of interest, the signal-strength $\mu$, defined as:

1 $$\begin{aligned} \mu = \sigma (WH)\times \mathrm {BR}(H\rightarrow aa) \times \mathrm {BR}(a \rightarrow bb)^2, \end{aligned}$$

where $\sigma (WH)$ is the production cross section for $pp \rightarrow WH$.

Systematic uncertainties in the signal and background predictions (see Sect. 6) are accounted for in the likelihood function as a set of nuisance parameters, $\varvec{\theta }$. These parameters are implemented as Gaussian priors in the case of shape uncertainties and log-normal priors for uncertainties affecting the normalisation, with width parameters corresponding to the size of the respective uncertainties. Statistical uncertainties in the background estimates in each bin of the discriminant distributions are also taken into account via dedicated nuisance parameters in the fit.

**Table 2:** Summary of the impact of the considered systematic uncertainties (in %) on the normalisation of the signal ($m_a$ = 60 GeV) and the main backgrounds for the (4j, 4b) channel after the fit. The total uncertainty can differ from the sum in quadrature of individual sources due to correlations between them

Systematic uncertainty [%]	*WH*, $H \rightarrow 2a \rightarrow 4b$	$t\bar{t}$ + light	$t\bar{t} + c\bar{c}$	$t\bar{t} + b\bar{b}$
Luminosity	4	4	4	4
Lepton efficiencies	1	1	1	1
Jet efficiencies	6	4	4	4
Jet energy resolution	5	1	3	1
Jet energy scale	4	2	4	3
*b*-tagging efficiency	17	5	5	9
*c*-tagging efficiency	1	6	12	4
Light-jet-tagging efficiency	2	29	5	3
Theoretical cross sections	–	5	5	5
$t\bar{t}$: modelling	–	6	45	26
$t\bar{t}$+HF: normalisation	–	–	35	18
$t\bar{t}$+HF: modelling	–	–	–	5
Signal modelling	7	–	–	–
Total	21	31	54	21

The background-only hypothesis is tested by fitting the background predictions to the observed data, setting $\mu =0$ and maximising the likelihood over $\varvec{\theta }$. The best-fit $\mu$ is obtained by performing a binned likelihood fit to the data under the signal-plus-background hypothesis, i.e. maximising the likelihood function $L(\mu ,\varvec{\theta })$ over $\mu$ and $\varvec{\theta }$. The nuisance parameters $\varvec{\theta }$ allow variations of the predicted signal and background according to the corresponding systematic uncertainties, and their fitted values correspond to the deviations from the nominal predictions that globally provide the best fit to the data. This procedure allows a reduction of the impact of systematic uncertainties on the search sensitivity by taking advantage of the highly populated background-dominated channels included in the likelihood fit.

## Systematic uncertainties

Several sources of systematic uncertainty are considered that affect the normalisation or the shape of the signal and background contributions to the final discriminant distributions. Each source of systematic uncertainty is considered to be uncorrelated with other sources, but correlated across processes and channels where appropriate. This section describes the sources of systematic uncertainty considered in this search.

**Luminosity and pile-up** The uncertainty in the integrated luminosity is $5\,\%$, affecting the overall normalisation of all processes estimated from the simulation. It is derived, following a methodology similar to that detailed in Ref. [94], from a calibration of the luminosity scale using *x*–*y* beam-separation scans performed in August 2015. The uncertainty associated with the modelling of pile-up arises mainly from differences between the expected and observed fraction of the visible *pp* cross section.

**Reconstructed objects** Uncertainties associated with leptons arise from the reconstruction, identification and trigger efficiencies, as well as lepton momentum scales and resolutions. These efficiencies are measured using tag-and-probe techniques on $Z\rightarrow \ell ^+\ell ^-$ data and simulated events. The small differences found are corrected in the simulation. Negligible uncertainties arise from the corrections applied to adjust the lepton momentum scales and resolutions in simulation to match those in data. The combined effect of all these uncertainties results in an overall normalisation uncertainty in the signal and background of less than $1\,\%$.

**Fig. 5:**
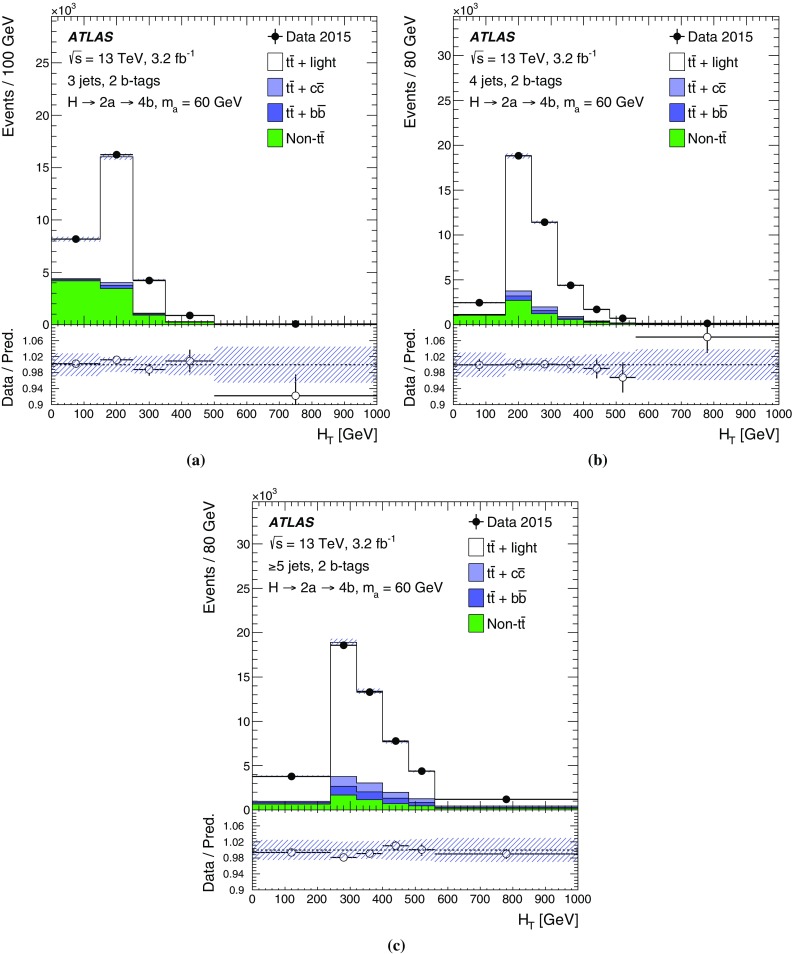
Comparison between the data and prediction for the distribution of the $H_\mathrm{T}$ variable used in the control regions with two *b*-tagged jets. These distributions are after the fit is performed on data under the background-only hypothesis. The *hashed area* represents the total uncertainty in the background. The last bin contains the overflow

**Fig. 6:**
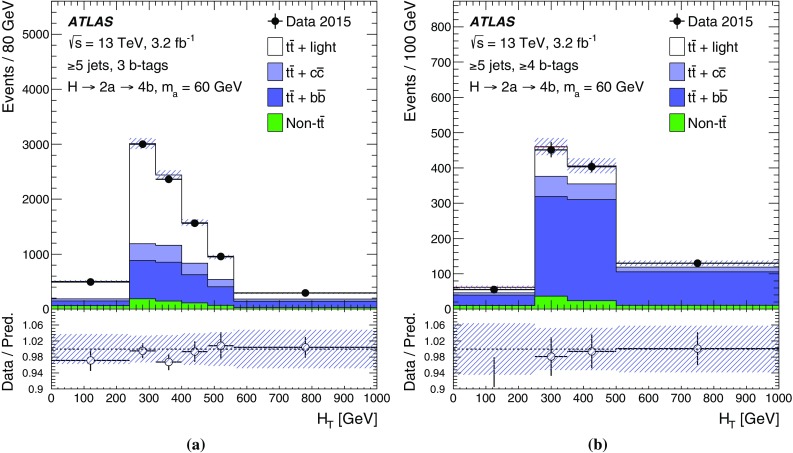
Comparison between the data and prediction for the distribution of the $H_\mathrm{T}$ variable used in the control regions with three and four *b*-tagged jets. These distributions are after the fit is performed on data under the background-only hypothesis. The last bin contains the overflow

**Fig. 7:**
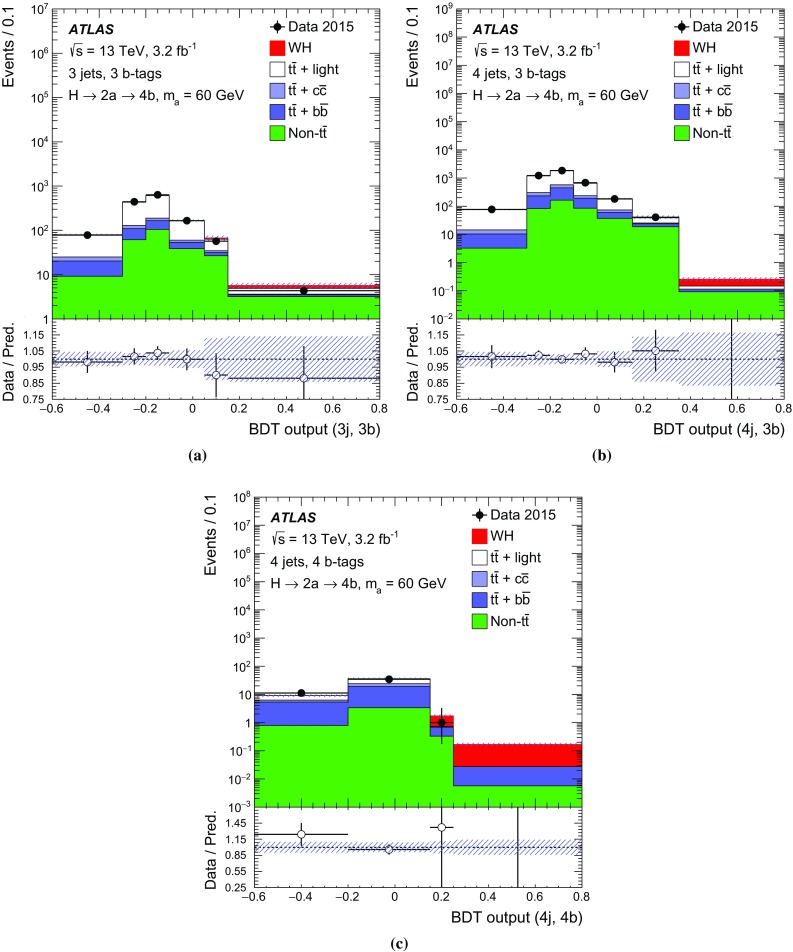
Comparison between the data and prediction for the distribution of the BDT discriminant used in the signal regions. These distributions are after the fit is performed on data under the background-only hypothesis. The *hashed area* represents the total uncertainty in the background. The distributions for the signal model (*WH*, $H\rightarrow 2a \rightarrow 4b$), with $m_a=60\,\mathrm{{GeV}}$, are normalised to the SM $pp \rightarrow WH$ cross section, assuming $\mathrm {BR}(H\rightarrow aa) \times \mathrm {BR}(a \rightarrow bb)^2 = 1$. The BDT output is determined in the range $[-1,1]$. The first and last bin contain the underflow and overflow, respectively. Markers are not drawn if they are outside the *y*-axis range

Uncertainties associated with jets arise from the efficiency of jet reconstruction and identification, as well as the jet energy scale and resolution. The largest contribution comes from the jet energy scale uncertainty, which depends on jet $p_{\text {T}}$ and $\eta$. It affects the normalisation of signal and backgrounds by approximately $5\,\%$ in the most sensitive search channels. Uncertainties associated with energy scales and resolutions of leptons and jets are propagated to $E_{\text {T}}^{\text {miss}}$. An uncertainty in the contribution from charged-particle tracks is also included in the $E_{\text {T}}^{\text {miss}}$ uncertainty [51]. Additional uncertainties originating from the modelling of the underlying event are negligibly small.

Several uncertainties are associated with the identification of the jet flavour, in particular the modelling of the *b*-, *c*-, and light-jet-tagging efficiencies in the simulation, which are corrected to match the efficiencies measured in data [47–49]. These uncertainties are derived from studies performed with data at $\sqrt{s} = 8 \mathrm{{TeV}}$ and are extrapolated to $13 \mathrm{{TeV}}$. They depend on the jet $p_{\text {T}}$ and the light-jet-tagging additionally depends on the jet $\eta$. The sources of systematic uncertainty in the tagging efficiencies are taken as uncorrelated between *b*-jets, *c*-jets, and light-jets. They have their largest impact in the (4j, 4b) channel, resulting in $4\,\%$ uncertainty in the $t\bar{t}$ background normalisation associated with the uncertainty in the *b*-jet-tagging scale factors, $8\,\%$ uncertainty in the $b\bar{b}$ background normalisation associated with the uncertainty in the *c*-jet-tagging scale factors, and $45\,\%$ uncertainty in the normalisation of the $t\bar{t}$+light background normalisation associated with the uncertainty in the light-jet-tagging scale factors.

**Background modelling:** Several sources of systematic uncertainty affecting the modelling of $t\bar{t}$+jets are considered. An uncertainty of approximately 6 % is assumed for the $t\bar{t}$ production cross section [72], including contributions from variations of the factorisation and renormalisation scales, and uncertainties arising from the PDFs, $\alpha _\mathrm{S}$, and the top-quark mass.

A 50 % uncertainty is assigned to the normalisation of the $t\bar{t}$ background. This uncertainty is derived from a comparison of the $t\bar{t}$ production cross sections predicted by Powheg-Box+Pythia and by Sherpa+OpenLoops at NLO (see Sect. 4) [33]. An additional $50\,\%$ uncertainty is assigned to the component of the $t\bar{t}$ background that contains exactly one *b*-hadron not originating from a top-quark decay matched to a particle jet. The same systematic uncertainty of $50\,\%$ is applied to the normalisation of the $b\bar{b}$ background in the absence of an NLO prediction for this process. The uncertainties in the $t\bar{t}$ components and $b\bar{b}$ are treated as uncorrelated.

Systematic uncertainties affecting the shape of the $t\bar{t}$ background account for the choice of generator, the choice of parton shower and hadronisation models, and the effects of initial- and final-state radiation. The uncertainties are derived from comparisons between the nominal simulation and alternative samples produced with Powheg-Box or MadGraph5_aMC@NLO interfaced to Pythia or Herwig ++ (see Sect. 4) and are treated as uncorrelated across $t\bar{t}$+jets backgrounds. Additional uncertainties are evaluated to account for the use of Sherpa+OpenLoops NLO to model the $t\bar{t}$ background. In particular, uncertainties are assessed for the PDFs, as well as the choice of shower recoil model and scale. An additional uncertainty accounts for limited knowledge of the component of the $t\bar{t}$ background originating from multiple parton interactions, which is not included in the NLO prediction. These systematic uncertainties are estimated following the methods described in Ref. [33].

**Table 3:** Expected event yields of the SM background processes in the three signal regions after performing the fit with the background-only hypothesis. The observed data and the number of expected signal events are also indicated. The signal yields are quoted for some representative values of $m_{a}$ and assume the SM $pp \rightarrow WH$ cross section, $\sigma _\mathrm{SM}(WH) = 1.37$ pb [57], and $\mathrm {BR}(H\rightarrow aa) \times \mathrm {BR}(a \rightarrow bb)^2 = 1$. The uncertainties include statistical and systematic components (systematic uncertainties are discussed in Sect. 6). The total uncertainty can differ from the sum in quadrature of individual sources due to correlations between them

Process	(3j, 3b)	(4j, 3b)	(4j, 4b)
$t\bar{t}$ + light	1089 ± 76	2940 ± 180	53 ± 16
$t\bar{t}$ + $c\bar{c}$	70 ± 28	280 ± 110	21 ± 11
$t\bar{t}$ + $b\bar{b}$	172 ± 55	610 ± 160	74 ± 15
$t\bar{t}$ +$\gamma /W/Z$	0.8 ± 0.1	4 ± 1	0.4 ± 0.1
*W* + jets	93 ± 31	129 ± 40	2 ± 1
*Z* + jets	18 ± 12	14 ± 10	–
Single-top-quark	135 ± 13	208 ± 17	8 ± 1
Multijet	48 ± 20	67 ± 28	4 ± 2
Dibosons	4 ± 1	9 ± 1	0.6 ± 0.4
$t\bar{t}$ + *H*	0.7 ± 0.1	4 ± 1	0.8 ± 0.2
Total	1640 ± 58	4270 ± 130	165 ± 15
Data	1646	4302	166
*WH*, $H \rightarrow 2a \rightarrow 4b$			
$m_{a} = 60\,\mathrm{{GeV}}$	10 ± 2	9 ± 1	3 ± 1
$m_{a} = 40\,\mathrm{{GeV}}$	11 ± 2	10 ± 2	2 ± 1
$m_{a} = 20\,\mathrm{{GeV}}$	6 ± 1	5 ± 1	0.7 ± 0.2

The uncertainties in the predictions for the total cross sections for the other background processes are applied as normalisation uncertainties and are: $5\,\%$ for each of the *W* / *Z*+jets and diboson processes, $+5\,\%$/$-4\,\%$ for single-top-quark production, $15\,\%$ for $t\bar{t} +\gamma /W/Z$ and $+9\,\%$/$-12\,\%$ for $t\bar{t} H$ [79–81, 95–99]. An additional uncertainty of $24\,\%$ is added in quadrature for each additional jet to account for the extrapolation to higher jet multiplicities, based on a comparison among different algorithms for merging LO matrix-element and parton shower simulations [100]. An uncertainty is applied to the modelling of the single-top-quark background to account for the choice of scheme to handle the overlaps between the $t\bar{t}$ and *Wt* final states. Small uncertainties arising from scale variations, which change the amount of initial-state radiation and thus the event kinematics, are also considered.

Uncertainties in the estimate of the multijet background come from the limited number of events in the data sample without the isolation requirement and from uncertainties in the measured non-prompt and prompt lepton efficiencies. The normalisation uncertainty assigned to this background is 60 %, as derived by comparing the multijet background prediction to data in control regions obtained by inverting the requirements on the $E_{\text {T}}^{\text {miss}}$ and on $m_{\mathrm T}^W$. An uncertainty in the shape of the predicted background distribution covers the difference between the prediction obtained by reducing the required number of *b*-tagged jets and the prediction at high *b*-tagged-jet multiplicity (see Sect. 4).

**Signal modelling** Several sources of systematic uncertainty affect the theoretical modelling of the signal acceptance. Uncertainties originate from the choice of PDFs, the factorization and renormalization scales, and the parton shower, hadronisation and underlying event models.

**Fig. 8:**
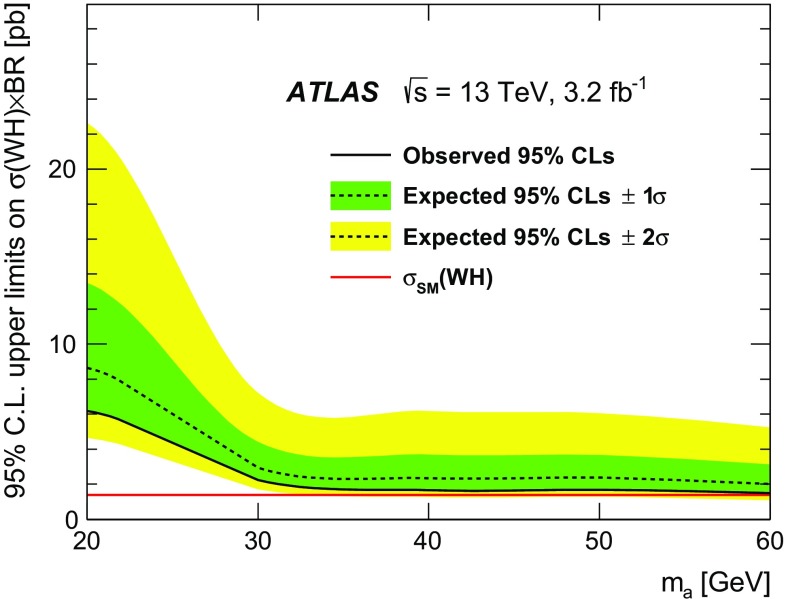
*Upper limit* at 95 % CL on $\sigma (WH)\times \mathrm {BR}$, where $BR=\mathrm {BR}(H\rightarrow aa) \times \mathrm {BR}(a \rightarrow bb)^2$, versus $m_a$. The observed $({\mathrm {CL}}_{\mathrm {s}})$ values (*solid black line*) are compared to the expected (*median*) $({\mathrm {CL}}_{\mathrm {s}})$ values under the background-only hypothesis (*dotted black line*). The surrounding *shaded bands* correspond to the 68 and 95 % CL intervals around the expected $({\mathrm {CL}}_{\mathrm {s}})$ values, denoted by $\pm 1\sigma$ and $\pm 2\sigma$, respectively. The *solid red line* indicates the SM $pp \rightarrow WH$ cross section, assuming $\mathrm {BR}(H\rightarrow aa) \times \mathrm {BR}(a \rightarrow bb)^2 = 1$. Markers are not drawn if they are outside the *y*-axis range

As described in Sect. 5.2, a binned maximum-likelihood fit is performed on the distributions of the final discriminant in the eight channels considered. The fit constrains systematic uncertainties from several sources thanks to the large number of events in the analysis channels considered and the variations in the background composition across channels. The channels with two *b*-tagged jets constrain the main uncertainties affecting the $t\bar{t}$+light background prediction, while the channels with $\ge$5 jets and $\ge$3 *b*-tagged jets are sensitive to the dominant uncertainties affecting the $t\bar{t}$+HF background prediction.

After performing the fit, the leading sources of systematic uncertainty are the modelling of the $t\bar{t}$+jets background and *b*-, *c*- and light-jet-tagging efficiencies. Table 2 summarises the systematic uncertainties by indicating their impact on the normalisation of the signal and the main backgrounds in the (4j, 4b) channel. The uncertainties for the other signal channels (3j, 3b) and (4j, 3b) are reduced to about $7\,\%$ for the $t\bar{t} +$light contribution, mainly due to the reduced dependence on the light-jet-tagging efficiency, and to about $12\,\%$ for the signal, primarily because of the reduced *b*-tagging efficiency uncertainty due to the lower *b*-tagged-jet multiplicity requirement.

## Results

The best fit of the background predictions to data in the binned maximum-likelihood fit is shown in Figures 5, 6 and 7. Table 3 shows the resulting yields and uncertainties for the signal regions after the fit. The SM background yields obtained after performing the fit are in agreement with the results from a fit using only the $H_{\text {T}}$ distributions in the control regions.

In the absence of a significant excess of data above the background prediction, upper limits are calculated for $\mu$, defined in Eq. (1). The modified frequentist method $({\mathrm {CL}}_{\mathrm {s}})$ [101] and asymptotic formulae [102] are used. Figure 8 shows the upper limits obtained at 95 % CL. The mass hypothesis $m_a$ is tested in steps of $10\,\mathrm{{GeV}}$ between 20 and $60\,\mathrm{{GeV}}$. The observed (expected) 95 % CL upper limits on $\mu$ range from 6.2 (8.6) pb, assuming $m_a = 20\,\mathrm{{GeV}}$, to 1.5 (2.0) pb, assuming $m_a = 60\,\mathrm{{GeV}}$. Assuming the SM $pp \rightarrow WH$ cross section, it is not possible to set limits on the branching fraction with the amount of data used. The reduced sensitivity for the light *a*-boson hypothesis is due to a lower acceptance caused by overlapping *b*-jets. The event yields indicated in Table 3 correspond to the sum of all BDT bins, while the fit is most sensitive in the highest BDT bins, where the data are slightly below the prediction, and hence the observed limit is slightly lower than the expected one.

## Conclusion

This paper presents a dedicated search for exotic decays of the Higgs boson to a pair of new spin-zero particles, $H \rightarrow aa$, where the new *a*-boson decays to *b*-quarks. The search focuses on the process $pp \rightarrow WH$ where the Higgs boson is produced in association with a $W$ boson. The analysis uses the *pp* collision dataset at $\sqrt{s} = 13 \mathrm{{TeV}}$ recorded by the ATLAS detector at the LHC in 2015, corresponding to an integrated luminosity of $3.2 \pm 0.2 {\text{ fb }^{-1}}$. The search for $H \rightarrow 2a \rightarrow 4b$ is performed in the mass range $20\,\mathrm{{GeV}} \le m_a \le 60\,\mathrm{{GeV}}$. The analysis uses several kinematic variables combined in a multivariate discriminant in signal regions and uses control regions to reduce the uncertainties in the backgrounds. No significant excess of data is observed relative to the SM predictions. Upper limits are derived for the product of the production cross section for $pp \rightarrow WH$ times the branching ratio for the decay $H \rightarrow 2a \rightarrow 4b$. The upper limit ranges from 6.2 pb for an *a*-boson mass $m_a = 20\,\mathrm{{GeV}}$ to 1.5 pb for $m_a = 60\,\mathrm{{GeV}}$.

## Footnotes

1 Throughout this paper, the symbol for a particle may be used to represent both the particle and its antiparticle.

2 ATLAS uses a right-handed coordinate system with its origin at the nominal interaction point (IP) in the centre of the detector and the *z*-axis coinciding with the axis of the beam pipe. The *x*-axis points from the IP to the centre of the LHC ring, and the *y*-axis points upward. Cylindrical coordinates (*r*,$\phi$) are used in the transverse plane, $\phi$ being the azimuthal angle around the beam pipe. The pseudorapidity is defined in terms of the polar angle $\theta$ as $\eta = - \ln \tan (\theta /2)$.

3 Particle jets are reconstructed by clustering stable particles, excluding muons and neutrinos, using the anti-$k_t$ algorithm with a radius parameter $R=0.4$. Muons and neutrinos are excluded to better reproduce the jet reconstruction based on calorimeter clusters.

4 The transverse mass is defined as $m_{\mathrm T}^W\equiv \sqrt{2 E_{\text {T}}^{\text {miss}} p_{\text {T}} ^{\ell } (1- \cos {\Delta \phi })}$, where $p_{\text {T}} ^{\ell }$ is the transverse momentum of the lepton and $\Delta \phi$ is the azimuthal angle between the lepton and $\vec {E}_{\text {T}}^{\text {miss}}$ directions.
